# DYRK1A promotes viral entry of highly pathogenic human coronaviruses in a kinase-independent manner

**DOI:** 10.1371/journal.pbio.3002097

**Published:** 2023-06-13

**Authors:** Madison S. Strine, Wesley L. Cai, Jin Wei, Mia Madel Alfajaro, Renata B. Filler, Scott B. Biering, Sylvia Sarnik, Ryan D. Chow, Ajinkya Patil, Kasey S. Cervantes, Clayton K. Collings, Peter C. DeWeirdt, Ruth E. Hanna, Kevin Schofield, Christopher Hulme, Silvana Konermann, John G. Doench, Patrick D. Hsu, Cigall Kadoch, Qin Yan, Craig B. Wilen

**Affiliations:** 1 Department of Immunobiology, Yale University School of Medicine, New Haven, Connecticut, United States of America; 2 Department of Laboratory Medicine, Yale University School of Medicine, New Haven, Connecticut, United States of America; 3 State Key Laboratory of Virology, Wuhan Institute of Virology, Chinese Academy of Sciences, Wuhan, Hubei Province, China; 4 Hillman Cancer Center, University of Pittsburgh Medical Center, Pittsburgh, Pennsylvania, United States of America; 5 Division of Infectious Diseases and Vaccinology, School of Public Health, University of California, Berkeley, Berkeley, California, United States of America; 6 University of Colorado Boulder, Boulder, Colorado, United States of America; 7 Department of Genetics, Yale University School of Medicine, New Haven, Connecticut, United States of America; 8 Department of Pediatric Oncology, Dana–Farber Cancer Institute and Harvard Medical School, Boston, Massachusetts, United States of America; 9 Broad Institute of MIT and Harvard, Cambridge, Massachusetts, United States of America; 10 Program in Virology, Harvard Medical School, Boston, Massachusetts, United States of America; 11 Genetic Perturbation Platform, Broad Institute of MIT and Harvard, Cambridge, Massachusetts, United States of America; 12 Department of Chemistry and Biochemistry, College of Science, The University of Arizona, Tucson, Arizona, United States of America; 13 Division of Drug Discovery and Development, Department of Pharmacology and Toxicology, College of Pharmacy, The University of Arizona, Tucson, Arizona, United States of America; 14 Department of Biochemistry, Stanford University School of Medicine, Stanford, California, United States of America; 15 Arc Institute, Palo Alto, California, United States of America; 16 Department of Bioengineering, University of California, Berkeley, Berkeley, California, United States of America; 17 Innovative Genomics Institute, University of California, Berkeley, Berkeley, California, United States of America; 18 Center for Computational Biology, University of California, Berkeley, California, United States of America; 19 Howard Hughes Medical Institute, Chevy Chase, Maryland, United States of America; 20 Department of Pathology, Yale School of Medicine, New Haven, Connecticut, United States of America; 21 Yale Cancer Center, Yale School of Medicine, New Haven, Connecticut, United States of America; Ulm University Medical Center, GERMANY

## Abstract

Identifying host genes essential for Severe Acute Respiratory Syndrome Coronavirus 2 (SARS-CoV-2) has the potential to reveal novel drug targets and further our understanding of Coronavirus Disease 2019 (COVID-19). We previously performed a genome-wide CRISPR/Cas9 screen to identify proviral host factors for highly pathogenic human coronaviruses. Few host factors were required by diverse coronaviruses across multiple cell types, but *DYRK1A* was one such exception. Although its role in coronavirus infection was previously undescribed, *DYRK1A* encodes Dual Specificity Tyrosine Phosphorylation Regulated Kinase 1A and is known to regulate cell proliferation and neuronal development. Here, we demonstrate that DYRK1A regulates *ACE2* and *DPP4* transcription independent of its catalytic kinase function to support SARS-CoV, SARS-CoV-2, and Middle East Respiratory Syndrome Coronavirus (MERS-CoV) entry. We show that DYRK1A promotes DNA accessibility at the *ACE2* promoter and a putative distal enhancer, facilitating transcription and gene expression. Finally, we validate that the proviral activity of *DYRK1A* is conserved across species using cells of nonhuman primate and human origin. In summary, we report that DYRK1A is a novel regulator of *ACE2* and *DPP4* expression that may dictate susceptibility to multiple highly pathogenic human coronaviruses.

## Abstract 2

This study reveals that Dual Specificity Tyrosine Phosphorylation Regulated Kinase 1A (DYRK1A) can facilitate coronavirus entry by driving chromatin accessibility and active transcription at coronavirus receptor gene loci, independent of its catalytic function.

## Introduction

Severe Acute Respiratory Syndrome Coronavirus 2 (SARS-CoV-2), the causative agent of Coronavirus Disease 2019 (COVID-19), is a beta coronavirus that has launched an ongoing pandemic that continues to threaten public health globally [1,2]. Two additional beta coronavirus family members (SARS-CoV and Middle East Respiratory Syndrome Coronavirus (MERS-CoV)) have caused more limited epidemics but with a higher case fatality rate [3–5]. There are also 4 endemic alpha and beta human coronaviruses that cause the common cold (HCoV-NL63, HCoV-OC43, HCoV-229E, and HCoV-HKU1). Despite zoonotic outbreaks of 3 beta coronaviruses in less than 20 years, our understanding of host factors that support these highly pathogenic human coronaviruses and the immune response to them remain incompletely understood [1,6,7].

The coronavirus life cycle commences with viral entry, which requires receptor binding and subsequent proteolytic processing of the viral spike (S) glycoprotein [8]. For initial infection and viral spread, a variety of epithelial cells can be targeted by SARS-CoV-2, but ciliated cells are the predominant target [9,10]. SARS-CoV and SARS-CoV-2 use the angiotensin-converting enzyme 2 (ACE2) receptor, whereas MERS-CoV engages dipeptidyl peptidase-4 (DPP4) as a receptor [11–15]. After receptor binding, the spike glycoprotein undergoes proteolytic cleavage at the cell surface by transmembrane serine protease 2 (TMPRSS2) or in the endosome by Cathepsin L (CTSL), enabling cell surface–mediated or endosomal entry, respectively [13,16–19]. Once internalized and proteolytically primed, the viral and host membranes fuse releasing the viral RNA genome and enabling viral protein translation and establishment of viral replication complexes [20–22]. Viral structural proteins (nucleocapsid, spike, membrane, and envelope) then package the nascent viral genome genomes into mature virions, which egress from the cell enabling viral spread [20–22].

We performed a genome-wide CRISPR/Cas9-based inactivation screen in African green monkey kidney Vero-E6 cells and in human lung epithelial Calu-3 cells [23,24]. Both Vero-E6 and Calu-3 cells are permissive to SARS-CoV, SARS-CoV-2, and MERS-CoV and endogenously express the cognate receptors ACE2 and DPP4 [23–27]. Our screens revealed several proviral genes shared by SARS-CoV, SARS-CoV-2, and MERS-CoV in both Vero-E6 and Calu-3 cells [23,24]. Other genome-wide CRISPR/Cas9 screens for SARS-CoV-2 and other related coronaviruses have since identified additional host dependency factors including proteins involved in viral entry, endocytic trafficking and sorting, cholesterol homeostasis, and autophagy [28–33]. While reproducibility between screens was excellent when performed on the same cell type, there was otherwise limited overlap across hits in these loss-of-function screens [28–33]. One notable exception was *DYRK1A*, which was a top enriched hit in both Vero-E6 and Calu-3 cells [23,24,34,35]. *DYRK1A* was not a hit in cell lines exogenously overexpressing ACE2, such as A549, Huh7.5, or HeLa cells [28–33]. Here, we sought to elucidate how DYRK1A regulates CoV infection and to determine the cell-type specificity of its function. Because DYRK1A was identified in cells with high endogenous ACE2 expression and was also a top hit for a replication competent SARS-CoV-2 pseudovirus, we posited that DYRK1A may function as a novel transcriptional regulator of coronavirus entry [23]. As viral entry is the first and rate-limiting step of infection, reducing viral entry can mitigate infection and pathogenesis [36–39]. While SARS-CoV-2 mRNA vaccines have proved highly efficacious at achieving this, the mechanisms that govern receptor expression represent a major gap in our knowledge of coronavirus biology [40].

*DYRK1A* encodes Dual Specificity Tyrosine Phosphorylation Regulated Kinase 1A, a member of the CMGC kinase group that includes cyclin-dependent kinases, mitogen-activated protein kinases, glycogen synthase kinases, and CDC-like kinases [41,42]. DYRK1A encodes a bipartite nuclear localization sequence and, as a result, accumulates predominantly in the nucleus, although cytosolic DYRK1A is reported in some contexts [43,44]. Located on chromosome 21 in the Down syndrome critical region (21q22.22), *DYRK1A* is a highly dosage-sensitive gene [45–47]. In Down syndrome (also known as trisomy 21), there is an extra copy of *DYRK1A*, resulting in overexpression that is strongly associated with disease pathogenesis and neurological developmental delay [48–52]. In contrast, down-regulated expression of *DYRK1A* can cause haploinsufficiency syndromes associated with microcephaly and autism spectrum disorder [53,54]. Interestingly, individuals with trisomy 21 are highly susceptible to SARS-CoV-2 with significantly elevated (5- to 10-fold) risk of infection, hospitalization, and death [54–59]. With such an increase in morbidity and mortality, Down syndrome may be among the top genetic disorders associated with the highest risk for COVID-19. The mechanisms underlying this increased COVID-19 morbidity and mortality are unknown.

DYRK1A shares notable identity with its *Drosophila* ortholog Minibrain and is highly conserved across lower to higher eukaryotes [41,60,61]. Regulation of DYRK1A is tightly controlled but poorly understood and modulates a myriad of functions including transcription, protein localization, protein–protein interactions, and proteolytic degradation [41,42,62–65]. Autophosphorylation of the DYRK1A tyrosine residue Y^321^ causes irreversible catalytic DYRK1A activation [42,64,66–68]. Once activated, DYRK1A functions as a mature serine/threonine kinase, with multiple phospho-substrates involved in cell cycle regulation, development, cell–cell signaling, and transcription, among others [41,42,61]. DYRK1A also has kinase-independent functions, including mRNA stabilization and transcriptional activation [69–73]. Mechanistic insights into these catalytically independent functions are lacking, but data suggest that DYRK1A may operate as a scaffold in these cases [44,70,74].

Despite its roles in many biological processes, the role of DYRK1A in viral pathogenesis remains incompletely explored. DYRK1A can promote human papillomavirus type 16 infection by phosphorylating the viral oncoprotein E7 [75]. Similarly, DYRK1A enables transformation by interacting with the oncoprotein E1A from human adenovirus type 5 (HAdV-5) [76]. Inhibition of DYRK1A kinase activity or genetic knockdown of DYRK1A also reduces human cytomegalovirus (HCMV) replication [77,78]. In the cases of both HCMV and HAdV-5, how DYRK1A performs this function is unclear. DYRK1A also possesses antiviral roles, such as by reducing HIV replication in vitro by down-regulating cyclin L2 and inhibiting long terminal repeat-driven transcription [79,80].

Here, we show that DYRK1A is critical for highly pathogenic human coronavirus infection in nonhuman primate and human cells, but not in mice. We demonstrate that DYRK1A is a novel regulator of coronavirus entry for both SARS-CoV, SARS-CoV-2, and MERS-CoV by promoting ACE2 and DPP4 receptor expression at the mRNA level. We further reveal that DYRK1A performs its proviral role in the nucleus independently of kinase function, suggesting a previously undescribed mechanism of DYRK1A activity in viral infection.

## Results

### DYRK1A promotes viral entry for SARS-CoV-2, SARS-CoV, and MERS-CoV

**Fig 1:**
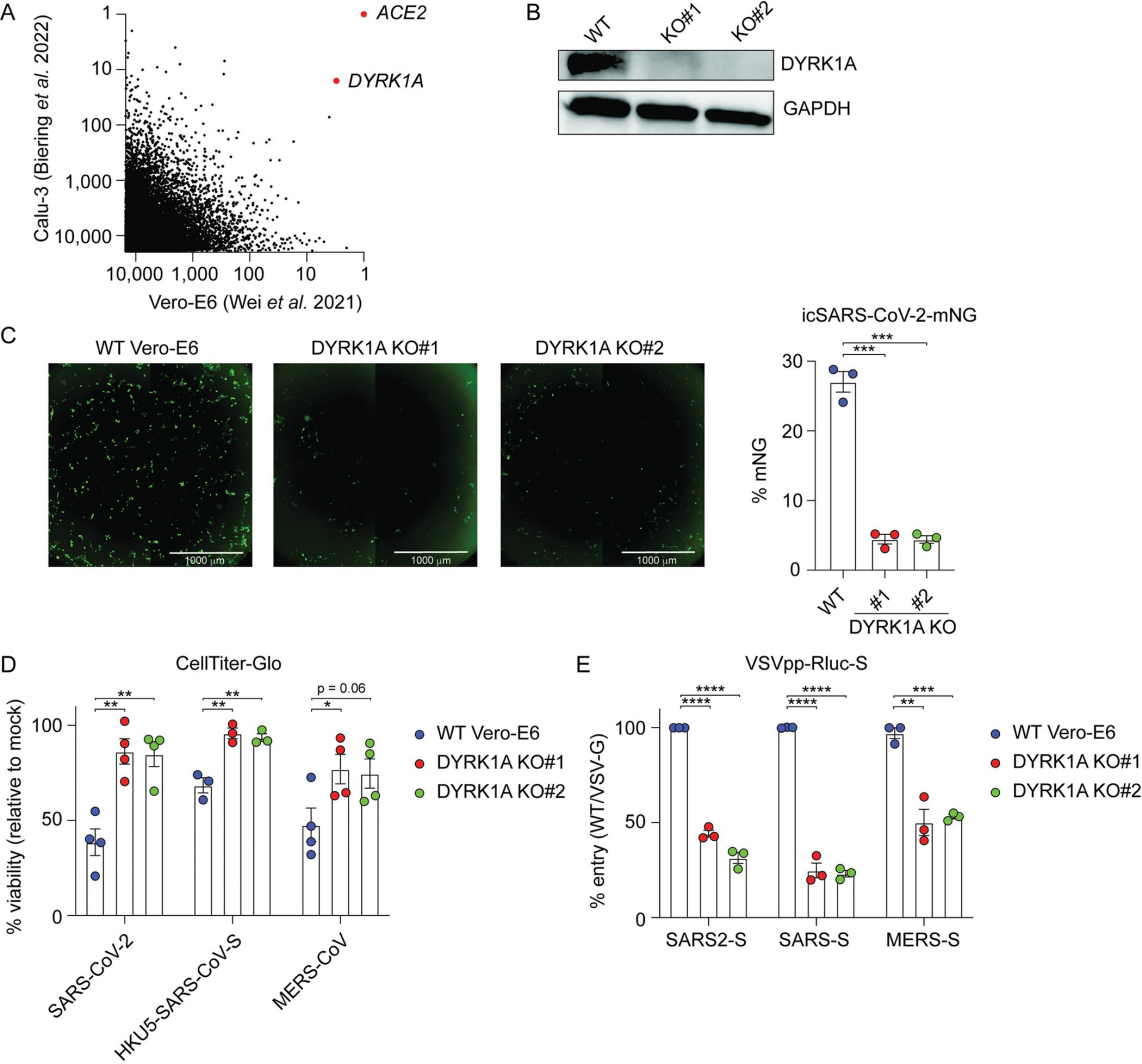
DYRK1A promotes viral entry for SARS-CoV, SARS-CoV-2, and MERS-CoV. **(A)** XY plot comparing the top 10,000 enriched genes that promote SARS-CoV-2 infection in genome-wide CRISPR screens performed in Vero-E6 cells (African green monkey kidney cells) [23] and Calu-3 cells (human lung epithelial cells) [24]. DYRK1A scored as the most strongly enriched gene after ACE2, supporting a conserved proviral role for DYRK1A in nonhuman primate and human cells. **(B)** Immunoblot for 2 single-cell monoclonal KOs of DYRK1A (KO#1 and KO#2) generated from parental Vero-E6 cells. **(C)** WT Vero-E6 cells and DYRK1A KO cells were infected with icSARS-CoV-2-mNeonGreen (mNG) at an MOI of approximately 1.0 and imaged at 24 hpi (left). mNeonGreen+ expressing cell frequency (%mNG) was quantified from stitched images (right). Scale bar: 1000 μm. **(D)** DYRK1A KO and WT Vero-E6 cells were infected with SARS-CoV-2, HKU5-SARS-CoV-S, or MERS-CoV at an MOI of approximately 1.0, and cell viability was assessed at 72 hpi with CellTiter-Glo. % viability was calculated relative to uninfected controls. **(E)** DYRK1A KO and WT Vero-E6 cells were infected with VSVpp encoding CoV spike proteins and an Rluc reporter at 24 hpi. % entry for VSVpp-Rluc-SARS2-S, VSVpp-Rluc-SARS-S, and VSVpp-Rluc-MERS-S was normalized to VSVpp-Rluc-VSV-G control and WT Vero-E6 cells. Data were analyzed by unpaired Student *t* test; * *p* < 0.05, ** *p* < 0.01, *** *p* < 0.001, **** *p* < 0.0001. Shown are means ± SEM. Data in **(C)**, **(D)**, and **(E)** are representative of 3 independent biological experiments performed with at least 3 technical replicates. Data underlying this figure can be found in S1 Data and S1 Raw Images. ACE2, angiotensin-converting enzyme 2; DYRK1A, Dual Specificity Tyrosine Phosphorylation Regulated Kinase 1A; hpi, hours postinfection; KO, knockout; MERS-CoV, Middle East Respiratory Syndrome Coronavirus; Rluc, Renilla luciferase; SARS-CoV-2, Severe Acute Respiratory Syndrome Coronavirus 2; VSVpp, VSV pseudovirus; WT, wild-type.

We and others recently identified DYRK1A as a critical host factor for SARS-CoV-2, MERS-CoV, and chimeric HKU5 (bat coronavirus) expressing the SARS-CoV spike (HKU5-SARS-CoV-S) in a genome-wide CRISPR/Cas9 inactivation screen in Vero-E6 cells [23,34]. In 2 additional independent genome-wide CRISPR screens in Calu-3 human immortalized lung cancer cells, DYRK1A was also identified as a top proviral gene for SARS-CoV-2 [24,34]. By comparing the top 10,000 enriched genes ranked by z-score for SARS-CoV-2 across Vero-E6 and Calu-3 cells, we identified *DYRK1A* as second most strongly enriched gene after only *ACE2* [23,24] (**Fig 1A**). In a separate screen, *DYRK1A* was the third most strongly enriched hit after *ACE2* [34]. We generated single-cell knockout (KO) clones of DYRK1A in Vero-E6 cells using CRISPR/Cas9 to validate screening results and clarify the proviral mechanism underlying DYRK1A activity in coronavirus infection (**Fig 1B**). We challenged clonal KO cells with an infectious clone of SARS-CoV-2 encoding the fluorescent reporter mNeonGreen (icSARS-CoV-2-mNG) and quantified viral infection by microscopy [81]. Cells deficient in DYRK1A were less susceptible to SARS-CoV-2 infection (**Fig 1C**). Consistent with this finding and our screen, loss of DYRK1A conferred a reduction in virus-induced cell death by SARS-CoV-2, HKU5-SARS-CoV-S, and MERS-CoV (**Fig 1D**). We next tested whether DYRK1A acts at viral entry using a pseudotype assay, where coronavirus spike proteins are expressed on a replication-deficient vesicular stomatitis virus (VSV) encoding a luciferase reporter. This pseudovirus enables a single round of spike-dependent viral entry. Cells lacking DYRK1A exhibited a significant defect in CoV pseudovirus entry with a magnitude similar to ACE2 and CTSL KO (**Figs 1E and S1**). These data highlight DYRK1A as a proviral host factor for highly pathogenic human coronaviruses, including SARS-CoV-2, which regulates spike-mediated viral entry in Vero-E6 cells.

### DYRK1A regulates expression of the receptors ACE2 and DPP4 at the transcript level

**Fig 2:**
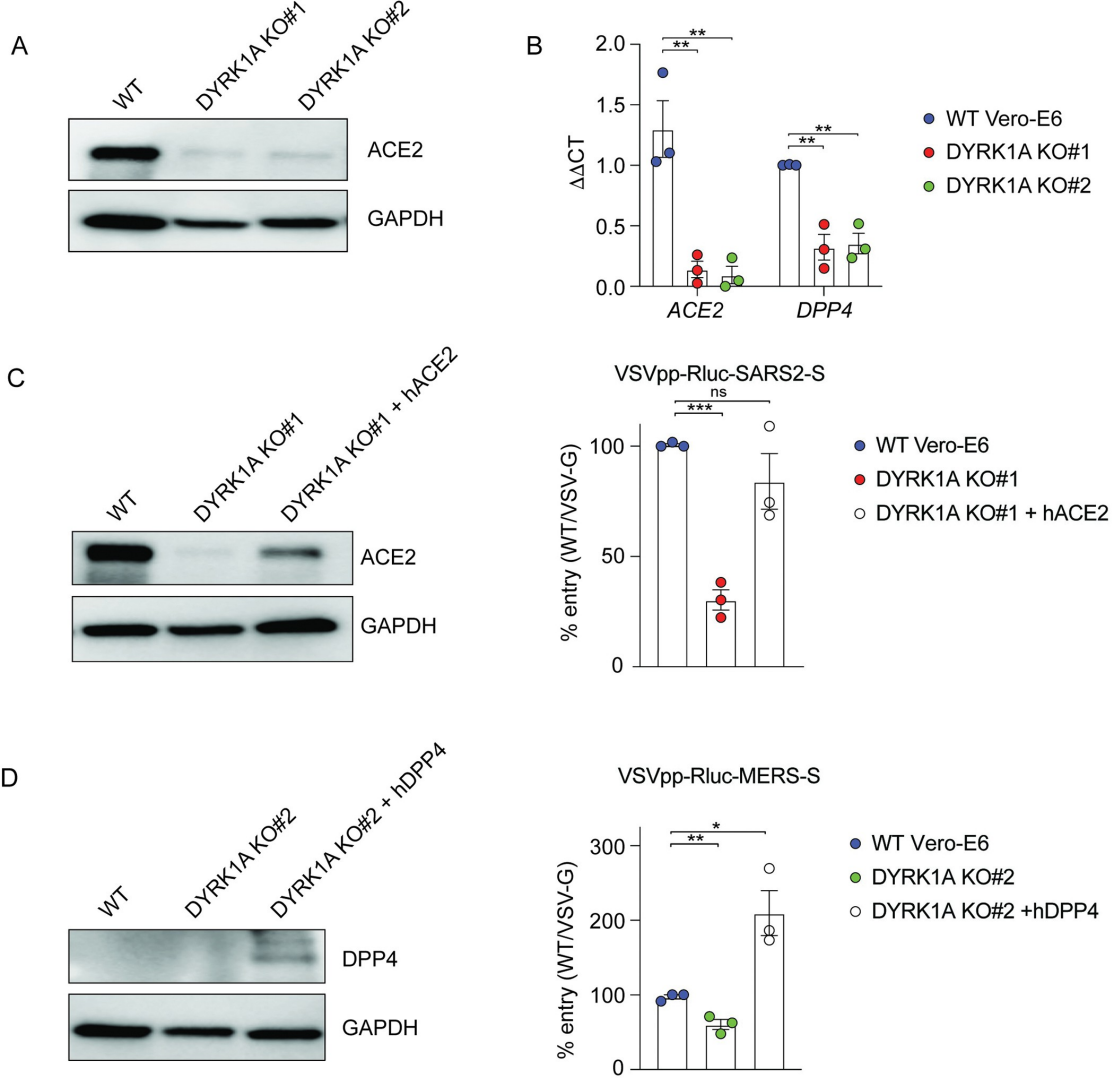
DYRK1A regulates expression of ACE2 and DPP4. **(A)** Immunoblot for ACE2 in WT Vero-E6 and DYRK1A KO cells. **(B)** mRNA abundance of *ACE2* and *DPP4* transcripts in WT Vero-E6 and DYRK1A KO clones assessed by qPCR. ΔΔCT values are calculated relative to actin and normalized to WT Vero-E6 values. **(C)** WT Vero-E6, DYRK1A KO#1, and DYRK1A KO#1 overexpressing recombinant hACE2 were infected with VSVpp-Rluc-SARS2-S, and % entry was assessed at 24 hpi. Immunoblot confirms rescue of ACE2 expression in DYRK1A KO#1. **(D)** WT Vero-E6, DYRK1A KO#2, and DYRK1A KO#2 overexpressing recombinant hDPP4 were infected with VSVpp-Rluc-MERS-S, and % entry was assessed at 24 hpi. Immunoblot confirms DPP4 overexpression in DYRK1A KO#2. % Entry in **(C)** and **(D)** was normalized to VSVpp-Rluc-VSV-G and WT Vero-E6 cells. Data were analyzed by unpaired Student *t* test; ns *p* > 0.05, * *p* < 0.05, ** *p* < 0.01, *** *p* < 0.001. Shown are means ± SEM. Each data point is the mean of 3–5 technical replicates. All experiments were performed at least 3 independent times. Data underlying this figure can be found in S1 Data and S1 Raw Images. ACE2, angiotensin-converting enzyme 2; DPP4, dipeptidyl peptidase-4; DYRK1A, Dual Specificity Tyrosine Phosphorylation Regulated Kinase 1A; hACE2, human ACE2; hDPP4, human DPP4; hpi, hours postinfection; KO, knockout; Rluc, Renilla luciferase; VSVpp, VSV pseudovirus; WT, wild-type.

Because coronavirus entry is receptor dependent, we next compared the expression of the receptors ACE2 and DPP4 in wild-type (WT) cells and DYRK1A KO cells. In DYRK1A KO cells, ACE2 expression is notably reduced at the protein level by western blot (**Fig 2A),** but endogenous DPP4 expression was below the limit of detection even in WT cells (**Fig 2D**, left). To determine whether DYRK1A regulates ACE2 or DPP4 expression at the mRNA level, we performed quantitative reverse transcription PCR (RT-qPCR) to quantify mRNA abundance (**Fig 2B**). Loss of DYRK1A causes a significant reduction in mRNA transcript levels of both *ACE2* and *DPP4*, suggesting that DYRK1A may transcriptionally regulate the *ACE2* and *DPP4* loci or that DYRK1A may modulate the mRNA stability of these transcripts (**Fig 2B**). We then overexpressed *ACE2* or *DPP4* in DYRK1A KO cells by stable overexpression to clarify whether post-entry or protease-dependent entry may also be regulated by DYRK1A (**Fig 2C** and **2D**). Overexpression of ACE2 and DPP4 both rescued the entry defect conferred by loss of DYRK1A for SARS-CoV-2 and MERS-CoV pseudoviruses, respectively (**Fig 2C** and **2D**). Taken together, these data indicate that DYRK1A promotes viral entry by supporting the expression of *ACE2* and *DPP4* at the mRNA level.

### The proviral role of DYRK1A is kinase independent in nature

**Fig 3:**
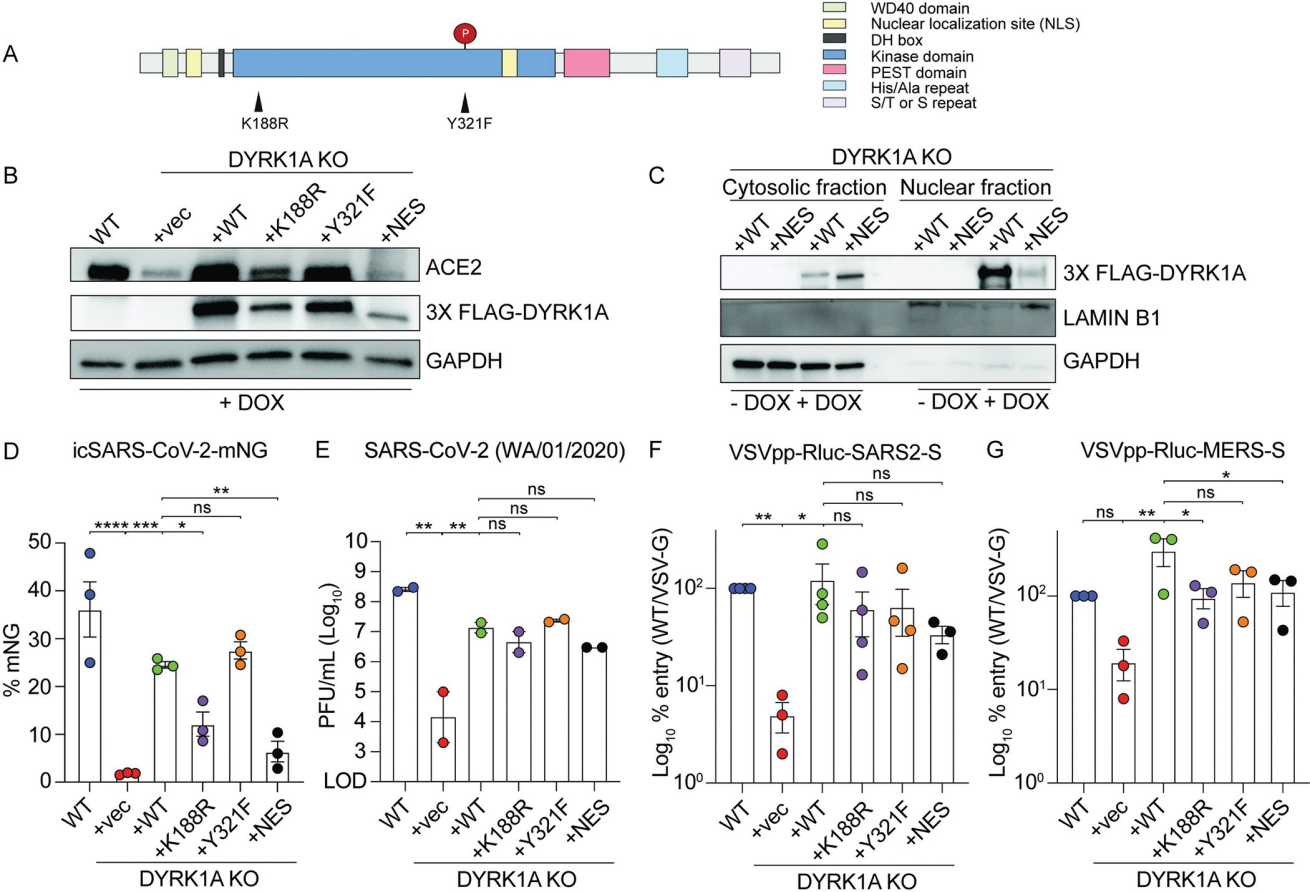
The proviral role of DYRK1A is kinase independent. **(A)** DYRK1A protein domains and engineered point mutations. Nuclear localization mutants were generated by deletion of the bipartite nuclear localization motif and addition of a C-terminal nuclear export signal. **(B)** Immunoblot for cells expressing an empty vector (KO+vec), WT DYRK1A (KO+WT), kinase dead DYRK1A (KO+K188R and KO+Y321F), or a nuclear localization mutant DYRK1A (KO+NES) were reintroduced to DYRK1A KO#1. Constructs were tagged with 3X FLAG and induced by DOX for 72 hours. Reintroduction of DYRK1A rescues ACE2 expression relative to WT Vero-E6 cells. **(C)** Immunoblot after cytosolic-nuclear fractionation of DYRK1A KO+WT or DYRK1A KO+NES, demonstrating that DYRK1A is predominantly localized to the nucleus until disruption of the bipartite nuclear localization motif and addition of a nuclear export signal. DYRK1A expression was induced by DOX for 72 hours prior to fractionation. **(D, E)** WT Vero-E6 cells, DYRK1A KO cells, and cells overexpressing DYRK1A after DOX induction for 72 hours were infected with **(D)** icSARS-CoV-2-mNeonGreen (mNG) at an MOI of approximately 1 and **(E)** SARS-CoV-2 WA/01/2020 at an MOI of approximately 0.1. Viral replication was assessed by **(D)** mNeonGreen+ expressing cell frequency (%mNG) in stitched images at 48 hpi or **(E)** plaque assay at 24 hpi. **(F, G)** Similarly, WT Vero-E6 cells and DYRK1A KO cells with reintroduced DYRK1A were infected with **(F)** VSVpp-Rluc-SARS2-S or **(G)** VSVpp-Rluc-MERS-S, and % entry was assessed at 24 hpi. % Entry was normalized to VSVpp-Rluc-VSV-G and WT Vero-E6 cells. Data were analyzed ordinary one-way ANOVA **(D, E, G)** or by Kruskal–Wallis (**F**); ns *p* > 0.05, * *p* < 0.05, ** *p* < 0.01, *** *p* < 0.001, **** *p* < 0.0001. Data shown are means ± SEM of **(D, F, G)** 5–10 technical replicates and **(E)** 2 biological replicates. Each experiment was performed at least 2 independent times. Data underlying this figure can be found in S1 Data and S1 Raw Images. ACE2, angiotensin-converting enzyme 2; DOX, doxycycline; DYRK1A, Dual Specificity Tyrosine Phosphorylation Regulated Kinase 1A; hpi, hours postinfection; KO, knockout; Rluc, Renilla luciferase; VSVpp, VSV pseudovirus; WT, wild-type.

To confirm the proviral phenotype and to elucidate the mechanism of DYRK1A proviral activity, we reintroduced DYRK1A into our single-cell KO clones (**Fig 1B**) by lentiviral transduction (**Fig 3**). We transduced the following constructs: WT DYRK1A, kinase-null DYRK1A (encoding K188R or Y321F inactivating point mutations), and nuclear localization mutant DYRK1A (encoding a nuclear export signal and disruption of the bipartite nuclear localization motif) (**Fig 3A**). DYRK1A requires ATP for catalytic activity. ATP binds with Lys^188^ and a K188R substitution blocks DYRK1A phosphorylation activity [66,67,82]. Disruption of Y^321^ autophosphorylation by mutating tyrosine to phenylalanine (Y321F) renders DYRK1A catalytically inactive by preventing kinase maturation [66,83–85]. Because sustained DYRK1A overexpression can cause cell cycle exit [86–88], we generated these constructs under the control of a doxycycline inducible Tet-on promoter. We first confirmed that these addbacks could rescue DYRK1A and ACE2 expression (**Fig 3B**). Consistent with previous literature, expression of DYRK1A-K188R is weaker relative to WT and DYRK1A-Y321F constructs, likely due to protein destabilization (**Fig 3B**) [82]. Complementation with WT DYRK1A restored ACE2 expression (**Fig 3B**). Unexpectedly, loss of kinase activity (K188R and Y321F) enabled at least partial rescue of ACE2 expression by western blot, whereas loss of nuclear localization did not elicit detectable ACE2 protein expression, despite partial retention of DYRK1A in the nucleus (**Fig 3B and 3C**). To confirm DYRK1A complementation could rescue infection with authentic virus, we challenged these cell lines with the icSARS-CoV-2-mNG reporter virus or WT SARS-CoV-2 WA/01/2020 to assess viral replication by mNG production and by plaque assay, respectively [81] (**Figs 3D, 3E, and S2A**). Consistent with protein expression data, WT and kinase-dead constructs significantly rescued infection in DYRK1A KO cells (**Figs 3D, 3E, and S2A**). The nuclear localization mutant DYRK1A also restored infection, possibly due to residual DYRK1A in the nucleus (**Figs 3C, 3E, and S2A**). Next, we tested whether full or partial restoration of DYRK1A and ACE2 expression could rescue viral entry using pseudovirus. In all cases where DYRK1A was reintroduced, DYRK1A KO clones exhibited full or partial rescue of SARS-CoV-2 and MERS-CoV viral entry, including catalytic and localization mutants (**Fig 3F and 3G**). Because genetic approaches indicated that DYRK1A kinase activity is dispensable for SARS-CoV-2 infection, we then validated these findings using an orthogonal pharmacologic approach. We inhibited DYRK1A with 4 type I ATP-competitive kinase inhibitors: harmine, INDY, DYR219, or DYR533 (**S2B Fig**) [89–93]. In contrast to a cathepsin L inhibitor, DYRK1A inhibitors did not mitigate virus-induced cell death at tolerated concentrations (**S2B Fig**). These data suggest that existing DYRK1A active site inhibitors are ineffective against SARS-CoV-2, which is consistent with kinase-independent regulation of infection (**S2B Fig**). Collectively, these data support that nuclear DYRK1A regulate receptor expression in a kinase-independent manner.

### DYRK1A drives ACE2 and DPP4 expression by altering chromatin accessibility

**Fig 4:**
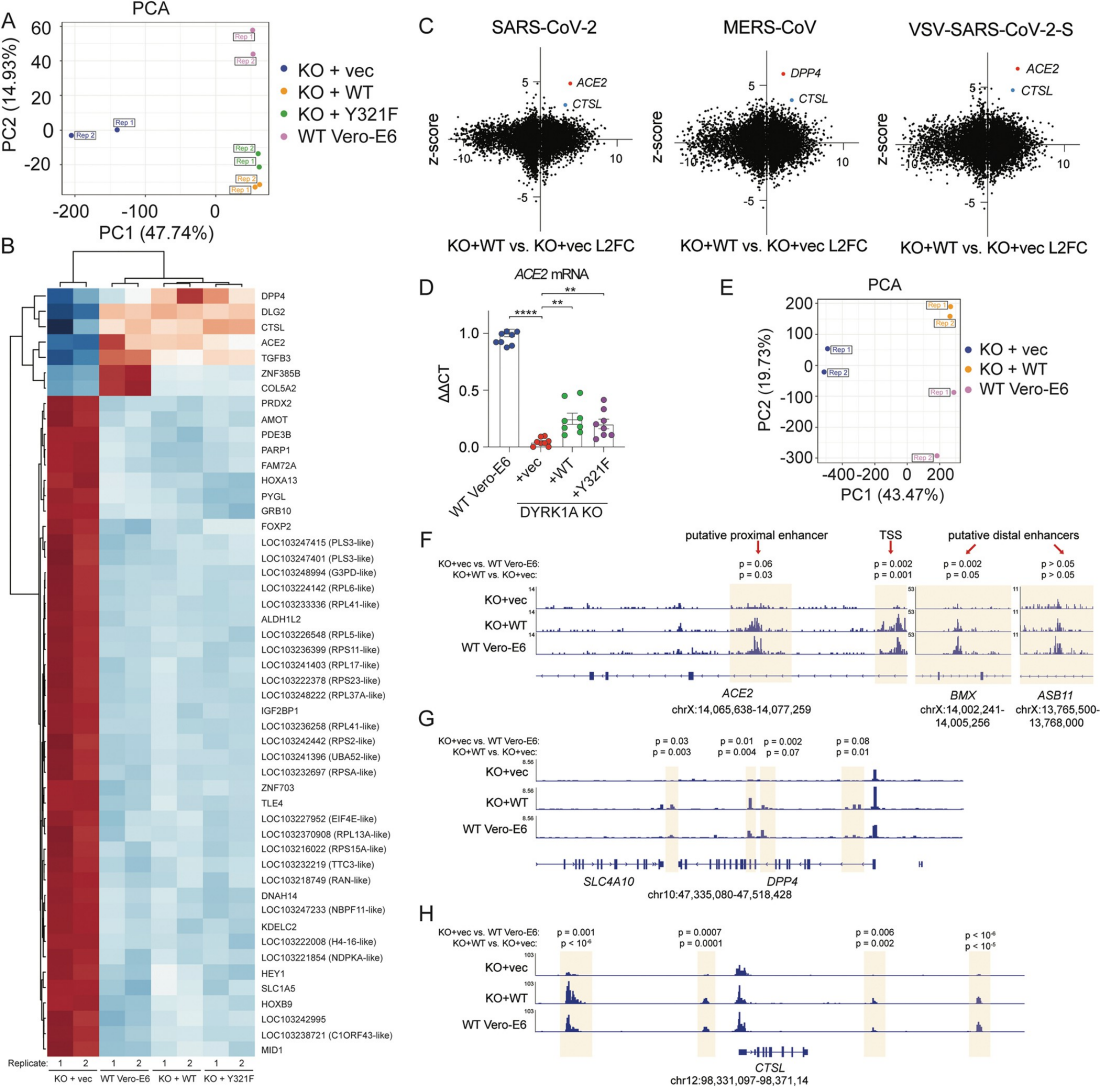
DYRK1A drives ACE2 and DPP4 expression by altering chromatin accessibility. **(A)** Principal component analysis of RNA-Seq experiments performed in WT Vero-E6, KO+vec, KO+WT, and KO+Y321F cells. Rep refers to independent biological replicates. **(B)** Heatmap depicting DEGs by RNA-Seq in WT Vero-E6, KO+vec, KO+WT, and KO+Y321F cells. **(C)** XY plot of RNA-Seq L2FC versus CRISPR z-scores in Vero-E6 cells [23] for SARS-CoV-2 (left), MERS-CoV (middle), or VSV-SARS-CoV-2-S (right). Denoted are receptor (*ACE2*, *DPP4*) and protease (*CTSL*) genes as significantly up-regulated proviral genes of interest. **(D)** RT-qPCR for *ACE2* validates RNA-Seq results and supports partial rescue of *ACE2* mRNA transcripts in cells where DYRK1A-WT or DYRK1A-Y321F are reintroduced. **(E)** Principal component analysis of ATAC-Seq experiments performed in WT Vero-E6, KO+vec, KO+WT, and KO+Y321F cells. Rep refers to independent biological replicates. **(F)** ATAC-Seq gene tracks for *ACE2*, highlighting increased accessibility at putative enhancers and near the TSS in the presence of DYRK1A. **(G)** ATAC-Seq gene tracks for *DPP4*, showing increased chromatin accessibility in the presence of DYRK1A. **(H)** ATAC-Seq genome tracks for *CTSL*, showing increased chromatin accessibility in the presence of DYRK1A. All experiments were performed in biological duplicate (RNA-Seq/ATAC-Seq) or triplicate (RT-qPCR). Data underlying this figure can be found in S1 Data and under GEO Accession GSE213999 (https://www.ncbi.nlm.nih.gov/geo/query/acc.cgi?acc=GSE213999). ACE2, angiotensin-converting enzyme 2; CTSL, Cathepsin L; DEG, differentially expressed gene; DPP4, dipeptidyl peptidase-4; DYRK1A, Dual Specificity Tyrosine Phosphorylation Regulated Kinase 1A; KO, knockout; L2FC, log_2_ fold-change; MERS-CoV, Middle East Respiratory Syndrome Coronavirus; RT-qPCR, quantitative reverse transcription PCR; SARS-CoV-2, Severe Acute Respiratory Syndrome Coronavirus 2; TSS, transcriptional start site; WT, wild-type.

Because DYRK1A can positively regulate transcription and gene expression [63,69,70,72,73], we next profiled global gene expression (RNA-Seq) and chromatin accessibility (ATAC-Seq) to assess the mechanism of DYRK1A-mediated regulation of *ACE2* and *DPP4* (**Figs 4 and S3**). Loss of DYRK1A resulted in up- or down-regulation of approximately 2,000 genes. Analysis of differentially expressed genes (DEGs) confirmed that loss of DYRK1A confers down-regulation of *ACE2* and *DPP4*, as well as *CTSL*, which encodes the protease for spike cleavage and viral entry in Vero-E6 cells [25] (**Figs 4B, 4C, and S3A**). Importantly, complementation with both DYRK1A-WT and DYRK1A-Y321F rescue expression of these genes (**Fig 4B and 4D**). There was no correlation between DYRK1A-dependent gene regulation and CRISPR genes (ranked by z-score) with the exception of *ACE2*, *DPP4*, and *CTSL* [23] (**Fig 4C**). Gene ontology analysis also revealed little overlap between biological pathways regulated by DYRK1A and those involved in SARS-CoV-2 infection (**S3B Fig**) [23]. Because RNA-Seq revealed that DYRK1A promotes increased mRNA levels for these genes, we next asked whether DYRK1A altered chromatin accessibility at these sites, thereby altering transcription. Loss of DYRK1A confers generally more open chromatin states across the genome (**S3C Fig**). In contrast, absence of DYRK1A resulted in reduced accessibility near the *ACE2* transcriptional start site (TSS), a putative proximal enhancer, and a putative distal enhancer situated within *BMX* (**Figs 4F and S3C**) [39]. Chromatin accessibility was restored at these sites upon reintroduction of DYRK1A (**Figs 4F and S3C**). A second putative distal enhancer located within *ASB11* does not seem to be altered by DYRK1A (*p* > 0.05), indicating that DYRK1A-mediated regulation of *ACE2* may be context or site specific (**Fig 4F**). Interestingly, three sites were identified where loss of DYRK1A led to significantly increased (*p* < 0.0005) chromatin accessibility within 5 kb of the *ACE2* TSS, suggesting that DYRK1A may also close off chromatin via repressive activity at the *ACE2* locus (**S3C Fig**). In comparison to SMARCA4, another *ACE2* regulator we have identified, DYRK1A-mediated DNA accessibility significantly overlaps with SMARCA4 at approximately 1/3 of sites (*p* < 0.00001 by Fisher’s exact *t* test), suggesting that some pathways may be coregulated by the two (**S4D Fig**). However, DYRK1A-mediated DNA accessibility (correlation coefficient approximately 0.33) and gene expression output (correlation coefficient approximately 0.08) overall poorly correlate with those regulated by SMARCA4 (**S4C and S4E Fig**) [39]. Moreover, SMARCA4 promotes open chromatin states at both putative distal enhancers (*BMX* and *ASB11*) and the putative *ACE2* proximal promoter [39]. Together, these data suggest that DYRK1A regulates the majority of DNA accessibility and gene expression independently of SMARCA4. Therefore, our data support that DYRK1A is a critical regulator of *ACE2* chromatin accessibility and transcription by a novel mechanism. Although enhancer regions and other regulatory elements are not well defined for *DPP4* and *CTSL*, we observed increased chromatin accessibility at sites proximal and distal to the respective TSS for these genes, which was not the case for SMARCA4 at these sites (**Fig 4G and 4H**). Unlike in the case of *ACE2*, putative insulator regions (i.e., sites where loss of DYRK1A led to more open chromatin) were not identified for *DPP4* or *CTSL*. These data highlight that DYRK1A—albeit not a canonical epigenetic modifying enzyme or transcription factor—can dramatically alter chromatin accessibility to drive transcription of a proviral gene expression axis that promotes viral entry.

### DYRK1A is a conserved proviral factor for SARS-CoV-2 in human lung epithelial cells

**Fig 5:**
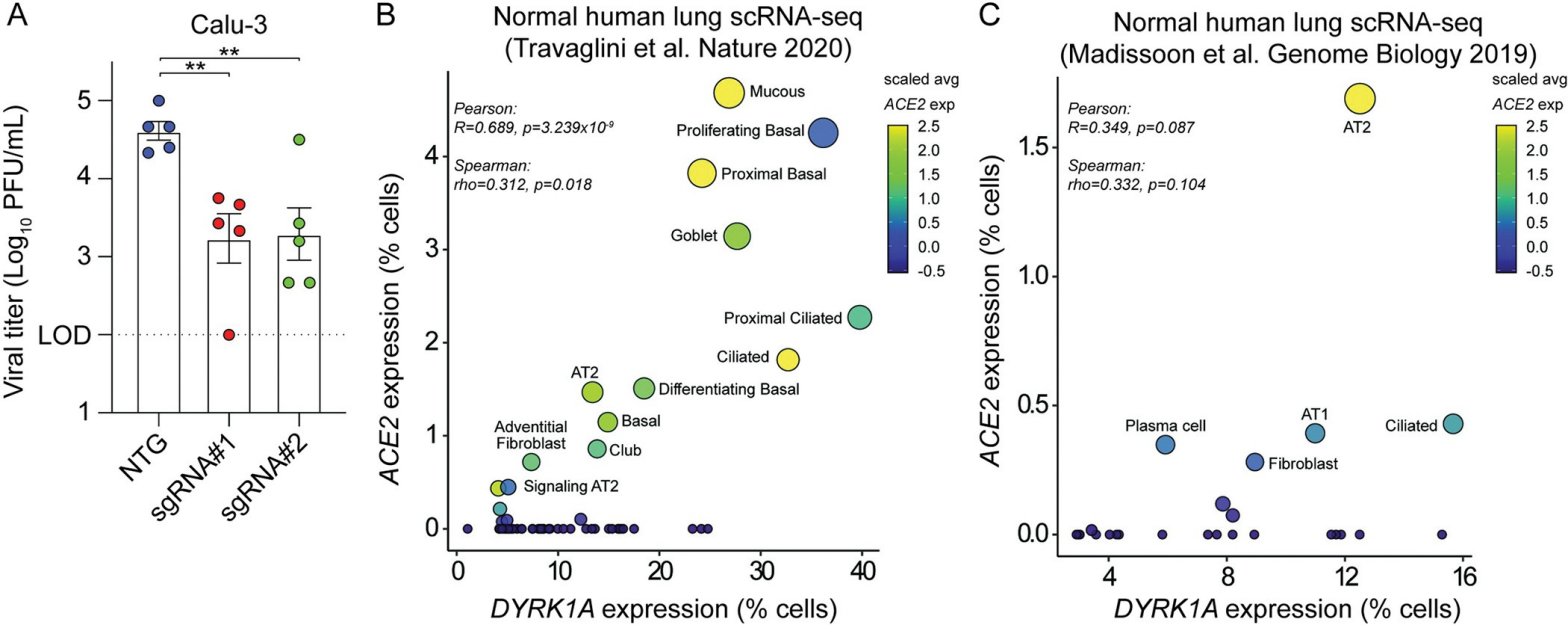
The proviral role of DYRK1A is conserved in human lung epithelial cells. **(A)** Polyclonal KOs of DYRK1A were generated in Calu-3 human lung epithelial cells with 2 independent guides (sgRNA#1 and sgRNA#2). Viral titers were assessed after 24 hpi by TCID50 and compared against an NTG. **(B, C)** Scatter plots correlating *DYRK1A* and *ACE2* mRNA levels in healthy human lung tissue [94,95]. Data in **(A)** are means ± SEM from 5 independent experiments. **(A)** Data were analyzed by unpaired Student *t* test; ** *p* < 0.01 or **(B, C)** Pearson and Spearman correlation statistical tests. Data underlying this figure can be found in S1 Data. ACE2, angiotensin-converting enzyme 2; DYRK1A, Dual Specificity Tyrosine Phosphorylation Regulated Kinase 1A; hpi, hours postinfection; KO, knockout; NTG, nontargeting guide.

We then sought to determine whether the proviral role of DYRK1A observed in Vero-E6 cells was conserved in human cells. We previously performed a subpool screen in human lung epithelial Calu-3 cells that identified DYRK1A as a positive regulator of SARS-CoV-2 infection [23]. To validate the results of that screen, we generated polyclonal KOs in Calu-3 cells using guides targeting *DYRK1A* or a nontargeting guide control (NTG). In Calu-3 cells, loss of *DYRK1A* causes a significant reduction in SARS-CoV-2 infection as quantified by tissue culture infectious dose (TCID50) assay (**Fig 5A**). Next, we assessed whether *DYRK1A* was coexpressed with *ACE2* and *DPP4* in healthy human lung tissue. Using existing RNA-Seq datasets, we identified that *DYRK1A* is indeed coexpressed with *ACE2* and *DPP4* (**Figs 5B, 5C, and S5**) [94,95]. Importantly, DYRK1A expression correlates with *ACE2* in epithelial cells such as AT2 cells, ciliated cells, and basal cells, which are major cellular targets of SARS-CoV-2 in vivo (**Figs 5B, 5C, and S5**) [9,10,94–96]. We recently showed that the mSWI/SNF complex regulates ACE2 chromatin accessibility and expression in human cells but not mice; therefore, we tested the species specificity for DYRK1A regulation of *Ace2* mRNA levels in mice [39]. Since loss of DYRK1A is embryonically lethal [97], we crossed an existing DYRK1A conditional deletion mouse (*Dyrk1a*^F/F^) to a tamoxifen-inducible Cre recombinase under control of the globally expressed ubiquitin c gene (*Ubc* CreERT2). We treated mice with tamoxifen for 5 days to conditionally ablate DYRK1A and then assessed *Ace2* mRNA levels. Loss of DYRK1A did not alter *Ace2* expression in the lung or distal small intestine **(S6 Fig)**. Overall, these findings suggest that DYRK1A is a critical host factor for SARS-CoV-2 infection that is conserved in primate cells.

## Discussion

A number of genome-wide CRISPR/Cas9 screens have been performed to unveil host factors that regulate SARS-CoV-2 infection [23,24,28–34,98]. We previously identified *DYRK1A* as a proviral gene for both SARS-CoVs and MERS-CoV in Vero-E6 cells and Calu-3 cells [23,24]. Additional recent independent screens in Vero-E6 and Calu-3 cells have confirmed our initial finding of *DYRK1A* as a host dependency factor for SARS-CoV-2 [34,35]. Numerous other SARS-CoV-2 genome-wide CRISPR KO screens failed to identify DYRK1A—a disparity likely attributable to their reliance on cells that ectopically overexpress ACE2. In such cells (A549-ACE2 and Huh7.5-ACE2), ACE2 regulation is uncoupled from the transcriptional regulators that promote endogenous ACE2 expression, rendering them nonessential for coronavirus infection [28–31]. As a result, numerous transcription factors and epigenetic regulators are obscured in screening results. Here, we demonstrate that DYRK1A supports transcription of *ACE2* and *DPP4* by altering chromatin accessibility, dictating susceptibility to highly pathogenic human coronaviruses across 2 mammalian species. However, we report that *Dyrk1a* fails to regulate *Ace2* mRNA levels in mice, which is concordant with our recent finding that epigenetic modulation of ACE2 fundamentally differs between mice and primates, revealing species-specific regulation of coronavirus receptor expression [39].

Using single-cell KO clones and addbacks, we report that nuclear DYRK1A promotes coronavirus entry by positively regulating *ACE2* and *DPP4* transcription via a kinase-independent mechanism. WT and kinase-null (DYRK1A-Y321F) complementation in vitro offered significant but partial rescue of live SARS-CoV-2 infection (approximately 70% to 80% of WT). Addbacks of another catalytically inactive mutant, DYRK1A-K188R, also exhibited partial but detectable rescue of SARS-CoV-2 infectivity. Of note, DYRK1A-K188R was not as effective at restoring icSARS-CoV-2-mNG infection relative to DYRK1A-Y321F. While this may result from minor residual kinase activity in DYRK1A-Y321F due to noncanonical autophosphorylation at Thr^111^, we anticipate that this is likely not the case due to equal rescue of viral entry by DYRK1A-Y321F and DYRK1A-K188R in both spike-dependent pseudovirus entry experiments and plaque assays [66,82,85]. Instead, we suggest that this difference is explained by lower expression of DYRK1A-K188R due to previously described instability of the DYRK1A-K188R mutant [82]. Notably, DYRK1A-NES also partially restored viral entry, likely due to incomplete displacement of DYRK1A to the cytosol.

Effective vaccines and direct acting antivirals including nirmatrelvir/ritonavir, remdesivir, and molnupiravir mitigate COVID-19, but novel therapeutic targets against current and future coronaviruses are needed to combat drug resistance and emerging viruses [99–105]. Although small molecule inhibitors of DYRK1A exist, these drugs are constrained by their limited selectivity and toxicity despite ongoing improvements in their design [106,107]. Moreover, these inhibitors structurally target the catalytic function of DYRK1A, which we have now shown to be dispensable for coronavirus entry. Therefore, consideration of DYRK1A as a therapeutic target will require new drug classes that are both tolerable and restrict DYRK1A activity independent of its catalytic function. Small molecules such as proteolysis targeting chimeras (PROTACs) that could degrade DYRK1A may offer such an approach [108,109]. To date, no DYRK1A-specific PROTACs have been generated, but selective degraders of DYRK1A such as CaNDY exist [110]. It was recently shown that SNX-544, an inhibitor of Hsp90, can greatly reduce SARS-CoV-2 infection in Vero-E6 and Calu-3 cells [111]. Inhibition of Hsp90 leads to destabilization and degradation of DYRK1A, suggesting that indirect mechanisms of DYRK1A depletion may exist that offer therapeutic benefit for SARS-CoV-2 [110].

Our current study further shows that DYRK1A promotes not only expression of *ACE2* and *DPP4*, but also *CTSL*. DYRK1A presence increases accessibility at recently identified putative *ACE2* regulatory elements (TSS, proximal enhancer, and a distal enhancer) and at sites that may regulate *DPP4* and *CTSL* [39]. As receptor and protease expression are critical for coronavirus entry, these findings suggest that DYRK1A operates as a critical modulator of chromatin accessibility and transcription to drive a proviral gene program that promotes viral entry. However, deletion of DYRK1A in Calu-3 cells—which lowly express CTSL and instead employ TMPRSS2 for spike proteolytic processing—still conferred increased resistance to SARS-CoV-2 infection. Nonetheless, we cannot preclude the possibility that DYRK1A may also regulate proteases or other aspects of viral entry besides receptor expression.

DYRK1A was previously reported to function as a positive regulator of transcriptional activity [63,69,70,72,73]. In the nucleus, DYRK1A can be recruited to enhancers and promoter regions, facilitating formation of the preinitiation complex or chromatin accessibility [69,70,72,112,113]. DYRK1A does not contain a DNA-binding domain and can bind and/or phosphorylate other proteins at these sites, supporting the function of other proteins such as RNA polymerase II (RNAPII) and histone acetyltransferases (P300/CBP) [70,112]. DYRK1A can also potentiate transcription independently of its kinase activity, such as by recruiting RNAPII to form the preinitiation complex or by binding known transcription factors as a scaffold, like androgen receptor-interacting protein 4 (ARIP4, also known as RAD54L2) and forkhead box O1 (FOXO1) [69,70,72]. Among the top proviral genes identified in our original CRISPR screen, many encode known DYRK1A interactors involved in transcriptional regulation, including those coding for the SWI/SNF complex (*SMARCA4*, *SMARCB1*, *ARID1A*, *SMARCE1*, *SMARCC1*, *DPF2)*, a histone methylase/demethylase complex (*KMT2D*, *KDM6A*), and transcriptional coregulators (*ARIP4/RAD54L2*) [23,44,63,114].

Because SMARCA4, KDM6A, and HMGB1 are known *ACE2* regulators, we asked whether DYRK1A may cooperate with one or more of these proteins, serving as a scaffold to promote *ACE2* or *DPP4* gene transcription [23,39,115]. Both HMGB1 and SMARCA4 alter chromatin accessibility at the *ACE2* locus, but how KDM6A modulates *ACE2* expression has yet to be defined [23,39]. DYRK1A–HMGB1 interactions have not been described, but DYRK1A can interact with SMARCA4 (the catalytic subunit of SWI/SNF) and SMARCB1 (a core subunit of SWI/SNF that stabilizes it and targets it to enhancers), resulting in phosphorylation of SMARCB1 [63,114,116]. Whether phosphorylation of SMARCB1 is critical for SWI/SNF activity is unclear, but SMARCA4 can rearrange nucleosomes at the *ACE2* promoter and 3 putative enhancers, driving accessibility and transcription of *ACE2* [39,117]. Overall, the DEGs and chromatin regions regulated by DYRK1A poorly correlate with those altered by HMGB1 or SMARCA4, although some pathways are shared between DYRK1A and SMARCA4 (**S4 Fig**) [23,39]. Furthermore, HMGB1 and SMARCA4 do not alter *DPP4* expression or promote MERS-CoV infection [23]. Together, these findings suggest that DYRK1A likely functions independently of HMGB1 and SWI/SNF to promote CoV infection. Still, we cannot exclude the possibility that SMARCA4 and DYRK1A may coordinate to coregulate some sites (i.e., *ACE2*) but not others (i.e., *DPP4*). Unlike SMARCA4 and HMGB1, the histone methyltransferase KDM6A regulates both SARS-CoV-2 and MERS-CoV infection [23,39,115]. KDM6A complexes with KMT2D, a histone demethylase, and the H3K27 acetyltransferase P300 and can activate enhancers [118]. DYRK1A can directly interact with KMT2D and P300, hyperphosphorylating P300 to drive H3K27 deposition and enhancer accessibility [44,112]. Because kinase activity is dispensable for DYRK1A-driven *ACE2* and *DPP4* expression, we suspect that DYRK1A also operates independently of KDM6A and KMT2D via a novel kinase-independent mechanism but cannot rule out that DYRK1A may act as a scaffold for these proteins. Clarifying the proteins that coordinate with DYRK1A, including transcription factor(s) that target DYRK1A to these sites, represent an important future direction.

ACE2 and DPP4 perform physiologic functions independent of their roles as coronavirus receptors. *ACE2* encodes a dipeptidyl carboxypeptidase that cleaves angiotensin I, maintaining homeostasis in the renin-angiotensin system [119]. Located on chromosome *Xp22*, *ACE2* is an X-linked, dosage-sensitive gene known to be transcriptionally regulated by EP300, SP-1, CEBP, GATA3, and HNF1/4 [119,120]. Recent efforts have aimed to identify novel *ACE2* modifiers, uncovering *PIAS1*, *SMAD4*, *BAMBI*, *KDM6A*, and *GATA6* [115,121]. *DPP4*, which is situated on chromosome *2q24*, encodes a serine exopeptidase that regulates glucose homeostasis [122,123]. The *DPP4* promoter contains binding sites for NF-kB, SP-1, EGFR, NF-1, STAT1, and HNF1 [124–126]. Until now, only hepatocyte nuclear factor (HNF) family proteins have been identified as shared transcriptional regulators for these genes. Here, we demonstrate for the first time that DYRK1A is a novel regulator for both genes. However, the impact of DYRK1A on the renin-angiotensin system and glucose homeostasis have yet to be thoroughly explored and warrant additional investigation. DYRK1A is also highly dosage sensitive, and whether fine-tuning of DYRK1A expression could alter these critical biological pathways is unknown.

In Down syndrome (trisomy 21), DYRK1A is overexpressed 1.5-fold due to its position on chromosome 21, and dysregulation of DYRK1A dosage directly contributes to neurological defects and disease [45–52]. Strikingly, Down syndrome significantly increases (5 to 10×) the risk of COVID-19 infection, hospitalization, and death relative to the general population [54–59]. While individuals with Down syndrome possess many comorbidities that could partially explain their predisposition to COVID-19 severity, together, they incompletely explain such an elevated risk. A recent network analysis revealed that *TMPRSS2*—which is also located on chromosome 21 and is subsequently overexpressed in Down syndrome—may contribute to COVID-19 severity by promoting increased viral entry [55,57]. Unlike TMPRSS2, DYRK1A does not directly interact with SARS-CoV-2, but we now show that DYRK1A positively regulates ACE2 expression and viral entry. Although *ACE2* is not globally overexpressed in Down syndrome, few tissues have detectable ACE2 expression at baseline and transcriptomic datasets for airway epithelial cells derived from individuals with Down syndrome are lacking [55]. Here, we show that DYRK1A deficiency (loss-of-function) leads to a significant reduction in ACE2 expression, but whether DYRK1A overexpression (gain-of-function) in Down syndrome is sufficient to up-regulate ACE2 in humans and subsequently contribute to COVID-19 severity remains to be seen and warrants additional investigation.

Our findings support that DYRK1A can promote DNA accessibility independently of its kinase activity, facilitating ACE2 and DPP4 expression for viral entry. We reveal that the proviral function of DYRK1A dictates susceptibility to diverse highly pathogenic human coronaviruses and is conserved across 2 mammalian species. While chemically targeting DYRK1A remains challenging and existing drugs will not suffice as viable therapeutics, our work highlights the value of considering genetic regulators or interactors in informed therapeutic target design.

## Materials and methods

### Cell culture

HEK293T (ATCC) and Vero-E6 (ATCC) cells were cultured in Dulbecco’s Modified Eagle Medium (DMEM, Gibco) with 5% heat-inactivated fetal bovine serum (FBS, VWR) and 1% Penicillin/Streptomycin (Gibco) unless otherwise noted. Vero-E6-ACE2-TMPRSS2 (gift from Barney Graham, NIH) were cultured with 5% DMEM, 5% FBS, 1% Penicillin/Streptomycin, and 5 μg/ml puromycin (Gibco). Calu-3 (ATCC) cells were cultured in RPMI 1640 (Gibco) with 1% Glutamax 100X (Gibco), 10% FBS, 1% Penicillin/Streptomycin, and 16 ng/ml hepatocyte growth factor (HGF, Stem Cell Technologies). When selecting cells transduced by lentivirus, Vero-E6 cells and Calu-3 were treated with 5 μg/ml or 1 μg/ml puromycin (Gibco), respectively. All cells were grown at 37°C in 5% CO_2_.

### Expression constructs, lentiviral packaging, and lentiviral transduction

All constructs were PCR amplified from a codon optimized gene block encoding the coding sequence of human DYRK1A (GenScript) using Q5 High-Fidelity DNA Polymerase with GC enhancer buffer (New England Biolabs). DYRK1A was cloned by Gibson assembly into pCW57.1-puro (gift of Katerina Politi, Yale School of Medicine). pCW57.1-DYRK1A K188R, Y321F, and NES constructs were generated by Q5 site-directed mutagenesis according to manufacturer’s instructions (New England Biolabs). All constructs were sequence validated by Sanger sequencing or Plasmidsaurus Oxford Nanopore sequencing. To generate lentiviral particles, a 3-component lentiviral system using 2.4 μg pVSV-G, 4.8 μg pSPAX2, and 8 μg lentiviral plasmid via calcium phosphate transfection was employed in 50% to 70% confluent HEK293T in a 10-cm dish. Supernatant containing lentivirus was harvested for 3 consecutive days and pooled. Cellular debris was clarified by centrifugation at 500 × *g*/5 minutes. For complementation, lentivirus was concentrated approximately 50-fold by using 4× PEGit lentiviral concentrator (MD Anderson). For lentiviral transduction, Vero-E6 or Calu-3 cells were transduced at 50% confluency and selected with 5 μg/mL (Vero-E6) or 2.5 μg/mL (Calu-3) puromycin 48 hours later. hACE2 and hDPP4 overexpressing lines were generated by stable lentiviral delivery of pLV-EF1a-ACE2-puro (gift of Akiko Iwasaki) and pLEX307-DPP4-puro (Addgene #158451) into *DYRK1A* KO clones.

### Viral stocks

Viral stocks were generated in Vero-E6 or Vero-E6-ACE2-TMPRSS2 cells seeded at approximately 80% confluency inoculated with HKU5-SARS-CoV-1-S (NR-48814), SARS-CoV-2 isolate USA-WA1/2020 (NR-52281), or MERS-CoV (icMERS-CoV EMC/2012) (NR-48813) from BEI resources at an MOI of approximately 0.01 for 3 days to generate a P1 stock. Vero-E6 or Vero-E6-ACE2-TMPRSS2 cells were inoculated with the P1 stock and incubated for 3 days or until 50% to 70% cytopathic effect was observed to generate a P2 stock. To generate icSARS-CoV-2-mNG stocks, lyophilized icSARS-CoV-2-mNG (World Reference Center for Emerging Viruses and Arboviruses, Galveston, TX) was resuspended in 500 μl deionized water and diluted 100-fold in medium. Diluted virus was added to 10^7^ Vero-E6 cells grown in T175 (Corning) for 3 days. All viral stock harvests were clarified by centrifugation (500 × g/5 minutes) and filtered through a 0.45-μm filter (Millipore Sigma), aliquoted, and stored at −80°C. All viral stocks were tittered by at least 2 independent plaque assays or TCID50 assays. Final viral stocks generated at Yale University were sequenced to confirm no mutations were generated during viral stock propagation. All work with infectious virus was performed in a Biosafety Level 3 facility in accordance to regulations and approval from the Yale University Biosafety Committee and Yale University Environmental Health and Safety.

### SARS-CoV-2 plaque assays

Vero-E6 cells were seeded at 4 × 10^5^ cells/well in 12-well plates (Corning) and were incubated overnight. The next day, media were removed and replaced with 100 μl of 10-fold serial dilutions of virus. Plates were incubated at 37°C for 1 hour, with gently rocking every 15 minutes to promote viral adherence. Wells were then covered with 1 mL overlay media (DMEM, 2% FBS, 0.6% Avicel RC-581) and incubated for 48 hours at 37°C. At 2 dpi, plates were fixed with 10% formaldehyde (Ricca Chemical) for 30 minutes and then stained with crystal violet solution (0.5% crystal violet (Sigma-Aldrich) in 20% ethanol) for 30 minutes. Crystal violet was aspirated and wells were rinsed with deionized water to visualize plaques.

### SARS-CoV-2 infections of polyclonal Calu-3 cells by TCID50

Polyclonal KOs of DYRK1A were generated in Calu-3 cells using 2 independent guides (sgRNA#1 and sgRNA#2). Calu-3 cells were seeded into 24-well plates at a density of 2 × 10^5^ cells/well. Two days later, cells were infected with SARS-CoV-2 post-seeding at an MOI of approximately 0.05. Viral inocula was incubated with cells for 30 minutes at 37°C. Unbound virus was aspirated and cells were washed once with 1× PBS. Cells were incubated for 24 hours and then subjected to mechanical lysis by freeze thaw. Infectious viral particles were tittered by serial dilution and incubated on 96-well plates coated with Vero-E6 cells. Each dilution was applied to 8 wells. Cytopathic effect was determined visually at 3 dpi, and TCID50/mL was calculated using the dilution factor required to produce CPE in half of the wells. Viral titers were assessed and compared against the NTG.

### SARS-CoV-2 fluorescent reporter virus assay

Cells were plated at 2,500 cells/well in a 384-well plate (Greiner) and adhered at 37°C for approximately 5 hours. icSARS-CoV-2-mNG was added at an MOI of approximately 1.0. Infection frequency was measured by mNeonGreen expression at 1 or 2 dpi by high-content imaging (Cytation 5, BioTek) configured with bright field and GFP cubes. Total cell numbers were determined from bright field images using Gen5 software. Object analysis was used to quantify the number of mNeonGreen-positive cells. Percent infection was calculated as the ratio between mNeonGreen+ cells and total cells.

### Generation of DYRK1A clonal Vero-E6 knockout and complemented cells

Vero-E6 DYRK1A KO cells were generated by lipofectamine transfection of Cas9-ribonucleoproteins (RNPs) CRISPR guide RNAs (gRNA) were synthesized by IDT (sequence: TCAGCAACCTCTAACCAACC). gRNAs were complexed in a 1:1 molar ratio with tracrRNA in nuclease-free duplex buffer by heating at 95°C for 5 minutes and then cooled to room temperature. Duplexes were combined with Alt-R Cas9 enzyme at room temperature for 5 minutes to form RNPs in Opti-MEM (Gibco) with 200 μl total volume. Complexes were mixed with RNAiMAX transfection reagent (Invitrogen) in Opti-MEM at room temperature for 20 minutes before transfection. Transfection was performed with 3.2 × 10^5^ Vero-E6 cells in suspension in a 12-well plate (Corning). Cells were incubated for 48 hours then stained with 1:500 Zombie Aqua (BioLegend) in 1× PBS (Gibco) prior to flow cytometry–based sorting on live cells. Single cells were sorted into 96-well plates (Corning). Clones were screened by increased resistance to rcVSV-SARS2-S virus-induced cell death, western blot, and were confirmed by Sanger sequencing. DYRK1A KO clones were complemented by lentiviral transduction of pCW57.1-puro vector containing full-length DYRK1A, kinase-dead DYRK1A (K188R or Y321F), or nuclear localization defective DYRK1A (NES) with an N-terminal 3× FLAG tag. Two days posttransduction, puromycin was added and cells were selected for 3 days to select for stably expressing addbacks. Stable DYRK1A expression was induced by the addition of 20 or 200 μg/ml doxycycline hyclate (Sigma-Aldrich) dissolved in DMSO (Sigma-Aldrich) for 24 or 72 hours. Expression of DYRK1A in complemented cells was confirmed by western blot for 3× FLAG and DYRK1A.

### Generation of DYRK1A polyclonal Calu-3 knockout cells

Oligonucleotides (Yale, Keck Oligo) were generated with BsmBI-compatible overhangs (guide 1 pair: CACCGTCAGCAACCTCTAACTAACC, AAACGGTTAGTTAGAGGTTGCTGAC; guide 2 pair: CACCGTGAGAAACACCAATTTCCGA, AAACTCGGAAATTGGTGTTTCTCAC). Oligos were annealed and phosphorylated using equimolar ratios of oligo pairs with 1× T4 Ligation Buffer (New England Biolabs) and T4 PNK (New England Biolabs) at 37°C for 30 minutes, 95°C for 5 minutes, then −5°C/minute to 25°C. LentiCRISPRv2 (Addgene #52961) was digested by BsmBI-v2 (New England Biolabs) for 2 hours at 55°C in 1× NEBuffer 3.1. Double-stranded oligonucleotides were ligated into the digested lentiCRISPRv2 vector using T4 DNA ligase (New England Biolabs) according to manufacturer’s protocol. Ligated vectors were transformed into Stbl3 cells and sequence verified for correct guide insertion. Lentiviral plasmids were cotransfected with packaging plasmids pSPAX2 and pVSV-G in HEK293T cells, and Calu-3 cells were transduced with lentiviral particles. Transduced cells were selected with puromycin for 2 weeks prior to infection.

### Pseudovirus production

VSV-based pseudotype viruses were generated as previously described in 10-cm dishes [127]. Briefly, HEK293T cells were transfected with pCAGGS or pCDNA3.1 vectors expressing the CoV spike (S) glycoprotein by calcium phosphate transfection and then inoculated with a replication-deficient VSV encoding Renilla luciferase in place of the G glycoprotein. After 1 hour at 37°C, unbound inoculum was removed and cells were washed with 4× PBS. Fresh media were added with anti-VSV-G clone I1 (8G5F11) (Absolute Antibody) to neutralize residual VSV-G virus [128]. After 24 hours, supernatant was harvested as viral stock and centrifuged at 3,000 rpm for 10 minutes to clarify cellular debris. VSVpp-SARS2-S stocks were concentrated using Amicon Ultra 100 kD filter columns (Millipore Sigma) at 3,000 rpm. VSVpp-SARS1-S and VSVpp-MERS-S stocks were not concentrated. All stocks were aliquoted and stored at −80°C. Plasmids encoding codon-optimized sequences of SARS-CoV-S and MERS-SΔCT were previously described [12,129]. Vector pCAGSS containing the SARS-CoV-2, Wuhan-Hu-1 S Glycoprotein Gene (NR-52310), was produced under HHSN272201400008C and obtained through BEI Resources, NIAID, NIH.

### Pseudovirus entry assay

Approximately 4 × 10^5^ Vero-E6 cells were seeded in 100 μl volume of each well of a black-walled clear bottom 96-well plate (Corning) and incubated at 37°C for approximately 5 hours to allow cells to adhere. VSV pseudovirus at 1:10 final concentration volume/volume for unconcentrated virus (VSVpp-SARS1-S or VSVpp-MERS-S) or 1:20 for concentrated virus (VSVpp-SARS2-S). Virus was incubated with cells for 24 hours, and cells were subsequently lysed with Renilla Luciferase Assay System (Promega) according to manufacturer’s instructions. Luciferase activity was measured using a microplate reader (BioTek Synergy). Pseudovirus entry was normalized to VSV-G within each condition, and percent entry was calculated relative to WT Vero-E6 cells after normalization.

### RT-qPCR

Total RNA was isolated from cells and lung homogenates using Direct-zol RNA MiniPrep Plus kit (Zymo Research), and 500 ng RNA was used for cDNA synthesis. cDNA synthesis was performed using random hexamers and ImProm-II reverse transcriptase (Promega). Quantitative PCR (qPCR) was carried out using 2 μl cDNA (diluted 1:10) with specific primers and probes (IDT) for African green monkey *b-actin* (Forward: 5′-GGATCAGCAAGCAGGAGTATG-3′; Reverse: 5′-AGAAAGGGTGTAACGCAACTAA-3′; Probe: /56-FAM/TCGTCCACC/ZEN/GCAAATGCTTCTAGG/3IABkFQ/), *ACE2* (Forward: 5′-AGAGGATCAGGAGTTGACATAGA-3′; Reverse: 5′-ACTTGGGTTGGGCACTATTC-3′; Probe: /56-FAM/ACCGTGTGG/ZEN/AGGCTTTCTTACTTCC/3IABkFQ/), and *DPP4* (Forward: 5′-GACATGGGCAACACAAGAAAG-3′; Reverse: 5′-GCCACTAAGCAGTTCCATCT-3′; Probe: /56-FAM/TTTGCAGTG/ZEN/GCTCAGGAGGATTCA/3IABkFQ/) genes. Reactions were prepared according to manufacturer’s recommendations for AmpliTaq Gold DNA polymerase (Applied Biosystems). For murine samples, cDNA was generated using 150 to 250 ng RNA. SYBR qPCR was performed using 2× Power SYBR Green (Applied Biosystems) with 5 μl cDNA (diluted 1:5) using the following mouse-specific primers: *b-actin* (Forward: ACTGTCGAGTCGCGTCCA; Reverse: ATCCATGGCGAACTGGTGG), *Ace2* (Forward: ACCTTCGCAGAGATCAAGCC, Reverse: CCAGTGGGGCTGATGTAGGA). All qPCR was performed using QuantStudio3 (Applied Biosystems). Data were analyzed by the ΔΔCT method, normalized to actin and control samples.

### Western blotting

Around 1 × 10^6^ cells were collected and lysed in Alfa Aesar Nonidet 40 (NP-40; 20 mM Tris–Hcl [pH 7.4], 150 mM NaCl, 1 mM EDTA, 1% Nonidet P-40, 10 mg/ml aprotinin, 10 mg/ml leupeptin, and 1 mM PMSF). Cell lysates were fractionated on SDS-PAGE pre-cast gels (BioRad) and transferred to a PVDF membrane by TurboTransfer (BioRad). Immunoblotting assays were performed with the following primary antibodies (1:1,000): anti-ACE2 (ProSci, cat#3217), anti-DYRK1A (Abcam, cat#ab259869), anti-FLAG (Sigma-Aldrich, cat#F3165), anti-DPP4 (R&D, cat#AF1180), anti-GAPDH (BioLegend, cat#649202), and anti-Lamin B1 (BioLegend, cat# 869801). Proteins were visualized with goat anti-mouse or goat anti-rabbit IgG secondary antibodies (1:5,000) diluted in 2% Omniblot milk (AmericanBio) in 1× TBST using a chemiluminescence detection system (BioRad ChemiDoc MP).

### SARS-CoV-2 in vitro DYRK1A inhibition assays

Harmine and INDY were purchased from Cayman Chemical, and DYR219 and DYR533 were synthesized in-house. Compounds were resuspended at a stock concentration of 40 to 50 mM in DMSO. Drugs were diluted 2-fold in DMSO and spotted into 384-well black skirted plates (Corning) in 20 nL at 1,000× drug stock using the Labcyte ECHO dispenser at the Yale Center for Molecular Drug Discovery. Approximately 1.25 × 10^3^ Vero-E6 cells were plated in total volume of 20 μl. Two days later, cells were infected with an MOI of approximately 1 SARS-CoV-2 isolate USA-WA1/2020 in 5 μl media. Cells were incubated for 3 days before assessing viability and virus-induced cell death by CellTiter-Glo according to manufacturer’s protocol (Promega). Luminescence was quantified using a plate reader (Cytation 5, BioTek). For each cell line, viability was determined in SARS-CoV-2–infected cells relative to uninfected cells.

### RNA-Seq

WT Vero-E6 cells, DYRK1A KO#1 + vector, and DYRK1A KO#1 + complements were seeded at 3 × 10^5^ cells/well in a 6-well plate and were treated with doxycycline for 72 hours to enable rescue of *ACE2* and *DPP4*. Samples were performed in biological duplicate and harvested by scraping. Total cellular RNA was extracted using the Direct-zol RNA MiniPrep Plus (Zymo Research), and libraries were prepared with rRNA depletion by the Yale Center for Genome Analysis. RNA-Seq libraries were sequenced on Illumina NovaSeq 6000 with a goal of at least 25 × 10^6^ reads per sample. Reads were aligned to reference genome chlSab2, NCBI annotation release 100 using STAR aligner v2.7.3a with parameters–winAnchorMultimapNmax 200 –outFilterMultimapNmax 100 –quantMode GeneCounts [130]. Differential gene expression was obtained using the R package DESeq2 v1.32 [131]. Heatmaps were generated using R package [132].

### ATAC-Seq

WT Vero-E6 cells, DYRK1A KO#1 + vector, and DYRK1A KO#1 + complements were seeded at 3 × 10^5^ cells/well in a 6-well plate and were treated with doxycycline for 72 hours to enable rescue of *ACE2* and *DPP4*. Samples were performed in biological duplicate and were harvested by scraping. Samples were submitted to Yale Center for Genome Analysis for library generation and were sequenced on an Illumina NovaSeq S4 instrument as 101 nt long paired-end reads with goal of at least 45 × 10^5^ reads per replicate. Reads were trimmed of Nextera adaptor sequences using Trimmomatic v0.39 [133] and aligned to chlSab2 using Bowtie2 v2.2.9 [134] with parameter -X2000. Duplicates were marked using Picard Tools v2.9.0 (Broad Institute. Version 2.9.0. “Picard Tools.” Broad Institute, GitHub repository. http://broadinstitute.github.io/picard/). Duplicated, unpaired, and mitochondrial reads were removed using SAMTools v1.9 [135]. Reads were shifted +4 bp and −5 bp for forward and reverse strands, respectively. Peaks were called using MACS2 v2.2.6 [136] with parameters–nomodel–keep-dup all -s 1 –shift 75 –extsize 150. Reads that fell inside peaks were counted using featureCounts v1.6.2 [137] and differential accessibility analysis was performed using DESeq2 v1.32 [131]. Bigwig files were generated using deeptools v3.1.3 with parameter–normalizeUsing RPKM [138]. Data were visualized with Integrated Genome Viewer.

### Generation of DYRK1A conditional knockout mice

*Dyrk1a*^*F/F*^ mice were obtained from the Jackson Laboratory (C57BL/6-*Dyrk1qa*^*tm1Jdc*^/J, Strain #027801) [139] and crossed to *Ubc*-Cre-ERT2 mice (B6.Cg*-Ndor*^*Tg(UBC-Cre/ERT2)1Ejb*^*/*1J, Strain #007001) also from the Jackson Laboratory [140]. Mice were genotyped using primer and probe sequences provided by Transnetyx (*Dyrk1a* Flox: Forward: 5′-TGTATGCTATACGAAGTTATTAGGTCCCT-3′, Reverse: 5′-CTTTTGTTAGTGTATGGCATAACTTGCA-3′, Reporter (FAM): 5′-CAGTGGGAGGATCCCCT-3′; *Ubc*-Cre-ERT2: Forward: 5′- AGGGCGCGCCGAATT-3′, Reverse: 5′- GGTAATGCAGGCAAATTTTGGTGTA-3′, Reporter (FAM): 5′-CCACCATGTCCAATTTA-3′.

### Analysis of existing single-cell RNA-Seq (scRNA-Seq) datasets from human lung tissue

Human lung scRNA-Seq datasets from healthy human lungs were accessed using the European Genome-phenome Archive (Accession EGAS00001004344) or the Human Cell Atlas Data Coordination Platform and NCBI BIOPROJECT (Accession code PRJEB31843) [94,95]. Data were downloaded and assessed for cell type–specific expression patterns using Seurat [141]. Prior to analyzing expression patterns, annotated doublets were removed. On a sample-by-sample basis, we quantified the number of *DYRK1A-*expressing cells from lung tissue based on the cell-type annotation by the authors. We then compared the % of cells expressing *ACE2* versus *DYRK1A* or *DPP4* versus *DYRK1A* and the scaled average expression. Data were statistically analyzed using Pearson and Spearman correlation tests.

### Ethics statement

The care and use of all animals were approved in accordance with the Yale Animal Resource Center and Institution Animal Care and Use Committee (#2021–20198) in agreement with the standards set by the *Animal Welfare Act*.

### Statistical analysis

All statistical analysis was performed in Prism GraphPad version 9.2.0 (San Diego, CA). Error bars indicate standard error of the mean unless otherwise indicated. Normally distributed data was analyzed using unpaired Student *t* tests while Mann–Whitney tests were performed for nonnormally distributed data. For more than 2 comparisons, ordinary one-way ANOVA or Kruskal–Wallis tests were performed according to normality. *P* values of <0.05 were considered statistically significant (*, *p* < 0.05; **, *p* < 0.01; ***, *p* < 0.001; ****, *p* < 0.0001).

## Supporting information

S1 Data Supplemental data containing raw summary data for all figures and statistics presented in the manuscript. (XLSX)

S1 Raw Images Raw images for western blot and microscopy data presented in the manuscript. (PDF)

S1 Fig Loss of ACE2 and CTSL reduce SARS-CoV-2 pseudovirus entry. Polyclonal deletion of *ACE2* or *CTSL* significantly reduce pseudotyped particles expressing the SARS-CoV-2 spike relative to WT Vero-E6 cells, like loss of *DYRK1A*. Cells were infected with VSVpp encoding CoV spike proteins and an Rluc reporter at 24 hpi. % Entry for VSVpp-Rluc-SARS2-S was normalized to VSVpp-Rluc-VSV-G control and WT Vero-E6 cells. Data were analyzed by unpaired Student *t* test; **** *p* < 0.0001. Shown are means ± SEM. Data are representative of 3 independent biological experiments performed with 3 technical replicates. Data underlying this figure can be found in S1 Data. ACE2, angiotensin-converting enzyme 2; CTSL, Cathepsin L; DYRK1A, Dual Specificity Tyrosine Phosphorylation Regulated Kinase 1A; hpi, hours postinfection; Rluc, Renilla luciferase; SARS-CoV-2, Severe Acute Respiratory Syndrome Coronavirus 2; VSVpp, VSV pseudovirus; WT, wild-type.
(PDF)

S2 Fig DYRK1A active site inhibitors do not block SARS-CoV-2 infection at tolerated concentrations. **(A)** WT Vero-E6 cells, DYRK1A KO cells, and cells overexpressing DYRK1A after DOX induction for 72 hours were infected with SARS-CoV-2 WA/01/2020 at an MOI of approximately 0.1 for 1, 24, or 48 hours. Plaque assays were performed in biological duplicate. Shown is 1 representative replicate. **(B)** WT Vero-E6 cells were treated with the positive control protease inhibitor calpain inhibitor III or a potent DYRK1A inhibitor (harmine, INDY, DYR219, and DYR533) for 48 hours. Cells were then infected with SARS-CoV-2 (MOI approximately 1), and cell viability was assessed 72 hpi via CellTiter-Glo. % Viability was calculated relative to uninfected or untreated controls. The half-maximal inhibitory concentration (IC50) and half-maximal cytotoxic concentration (CC50) were calculated for each drug using nonlinear regression dose response curves. Each experiment was performed at least 2 independent times. Shown are the means of 2–3 technical replicates. Data underlying this figure can be found in S1 Data. DOX, doxycycline; DYRK1A, Dual Specificity Tyrosine Phosphorylation Regulated Kinase 1A; hpi, hours postinfection; KO, knockout; SARS-CoV-2, Severe Acute Respiratory Syndrome Coronavirus 2; WT, wild-type.
(PDF)

S3 Fig DYRK1A regulates global gene expression and chromatin accessibility. **(A)** RNA-Seq volcano plots depicting DEGs in cells where DYRK1A is absent or reintroduced. **(B)** Enrichr pathway analysis for biological function enriched in the presence of DYRK1A. Gene set criteria included *p* < 0.05, L2FC<0 for KO+vec vs. WT Vero-E6 and L2FC>1.5 for both KO+WT and KO+Y321F vs. KO+vec. **(C)** ATAC-Seq volcano plots depicting DEGs in cells where DYRK1A is absent or reintroduced. All experiments were performed in biological duplicate**.** Data underlying this figure can be found under GEO Accession GSE213999. DEG, differentially expressed gene; DYRK1A, Dual Specificity Tyrosine Phosphorylation Regulated Kinase 1A; KO, knockout; L2FC, log2 fold-change; WT, wild-type.
(PDF)

S4 Fig DYRK1A and SMARCA4 share limited similarities in regulating chromatin accessibility and gene expression. **(A)** Principal component analysis of ATAC-Seq experiments performed in DYRK1A KO+vec, DYRK1A KO+WT, SMARCA4 KO+vec, and SMARCA4 KO+WT cells generated in a parental Vero-E6 background. Each experiment was performed in biological duplicate (replicates 1 and 2). DYRK1A and SMARCA4 loss share some molecular impacts, suggesting that some pathways may be coregulated (PC1), whereas others may be independently regulated (PC2). **(B)** Correlation heatmap comparing all sites from ATAC-Seq experiments in DYRK1A KO+vec, DYRK1A KO+WT, SMARCA4 KO+vec, and SMARCA4 KO+WT cells. **(C)** Correlation heatmap comparing chromatin accessibility by ATAC-Seq in DYRK1A or SMARCA4 complemented cells, identifying a correlation coefficient of 0.33 supporting approximately 33% of clusters may be correlated by DYRK1A and SMARCA4. **(D)** Venn diagram highlighting shared peaks gained by DYRK1A and SMARCA4 complementation. **(E)** Correlation heatmap comparing changes in RNA abundance in DYRK1A or SMARCA4 complemented cells, identifying a correlation coefficient of 0.08 supporting <10% of the top up-regulated/down-regulated genes are shared between DYRK1A and SMARCA4. **(F)** Gene set enrichment analysis from RNA-Seq experiments showing shared pathway regulation by DYRK1A and SMARCA4. Data underlying this figure can be found under GEO Accessions GSE213999 and GSE186201. DYRK1A, Dual Specificity Tyrosine Phosphorylation Regulated Kinase 1A; KO, knockout; WT, wild-type.
(PDF)

S5 Fig DYRK1A is coexpressed with ACE2 and DPP4 in human lung epithelial cells. **(A, B)** Scatter plots and **(C, D)** dot plots assessing scaled average expression of *DYRK1A and* **(A-E)** *DPP4* or **(C, D)** *ACE2* from existing scRNA-seq datasets [94,95]. Data in **(A. B)** were analyzed by Pearson and Spearman correlation statistical tests. ACE2, angiotensin-converting enzyme 2; DPP4, dipeptidyl peptidase-4; DYRK1A, Dual Specificity Tyrosine Phosphorylation Regulated Kinase 1A; scRNA-seq, single-cell RNA sequencing.
(PDF)

S6 Fig The regulatory role of DYRK1A on Ace2 expression is not conserved in mice. DYRK1A was conditionally deleted by treating *Dyrk1aA*^*F/F*^ Ubc CreERT2 mice with tamoxifen for 5 consecutive days. On day 6, **(A)** lung and **(B)** distal small intestine homogenates were assessed by RT-qPCR for *Ace2* expression. Each data point represents an individual mouse. Error bars represent ± SEM, and statistical comparisons were generated via Student *t* test; ns *p* > 0.05. Each experiment was performed 2 independent times with at least 3 mice per group. Shown the ΔΔCT values for each mouse normalized to actin and Cre negative controls. Data underlying this figure can be found in S1 Data. ACE2, angiotensin-converting enzyme 2; DYRK1A, Dual Specificity Tyrosine Phosphorylation Regulated Kinase 1A; RT-qPCR, quantitative reverse transcription PCR.
(PDF)

## Abbreviations

ACE2: angiotensin-converting enzyme 2
ARIP4: androgen receptor-interacting protein 4
COVID-19: Coronavirus Disease 2019
CTSL: Cathepsin L
DEG: differentially expressed gene
DMEM: Dulbecco’s Modified Eagle Medium
DPP4: dipeptidyl peptidase-4
DYRK1A: Dual Specificity Tyrosine Phosphorylation Regulated Kinase 1A
FBS: fetal bovine serum
FOXO1: forkhead box O1
gRNA: guide RNA
HAdV-5: human adenovirus type 5
HCMV: human cytomegalovirus
HGF: hepatocyte growth factor
HNF: hepatocyte nuclear factor
KO: knockout
MERS-CoV: Middle East Respiratory Syndrome Coronavirus
NTG: nontargeting guide
PROTAC: proteolysis targeting chimera
RNAPII: RNA polymerase II
RNP: ribonucleoprotein
RT-qPCR: quantitative reverse transcription PCR
SARS-CoV-2: Severe Acute Respiratory Syndrome Coronavirus 2
TMPRSS2: transmembrane serine protease 2
TSS: transcriptional start site
VSV: vesicular stomatitis virus
WT: wild-type

## References

[1] Hu B, Guo H, Zhou P, Shi Z-L. Characteristics of SARS-CoV-2 and COVID-19. Nat Rev Microbiol. 2020;19(3):141–154. doi: 10.1038/s41579-020-00459-7 33024307 PMC7537588

[2] Dong E, Du H, Gardner L. An interactive web-based dashboard to track COVID-19 in real time. The Lancet Infect Dis. 2020;20(5). doi: 10.1016/S1473-3099(20)30120-1 . 32087114 PMC7159018

[3] Guan Y, Zheng B, He Y, Liu X, Zhuang Z, Cheung C, et al. Isolation and characterization of viruses related to the SARS coronavirus from animals in southern China. Science. 2003;302(5643):276–278. Epub 2003/09/04. doi: 10.1126/science.1087139 . 12958366

[4] Drosten C, Kellam P, Memish Z. Evidence for camel-to-human transmission of MERS coronavirus. N Engl J Med. 2014;371(14):1359–1360. doi: 10.1056/NEJMc1409847 . 25271615

[5] Tang Q, Song Y, Shi M, Cheng Y, Zhang W, Xia X. Inferring the hosts of coronavirus using dual statistical models based on nucleotide composition. Sci Rep. 2015;5:17155. Epub 2015/11/26. doi: 10.1038/srep17155 ; PubMed Central PMCID: PMC4660426. 26607834 PMC4660426

[6] Weiss S. Forty years with coronaviruses. J Exp Med. 2020;217(5). doi: 10.1084/jem.20200537 . 32232339 PMC7103766

[7] Nelson C, Namasivayam S, Foreman T, Kauffman K, Sakai S, Dorosky D, et al. Mild SARS-CoV-2 infection in rhesus macaques is associated with viral control prior to antigen-specific T cell responses in tissues. Sci Immunol. 2022. doi: 10.1126/sciimmunol.abo0535 . 35271298 PMC8995035

[8] Hofmann H, Pöhlmann S. Cellular entry of the SARS coronavirus. Trends Microbiol. 2004;12(10). doi: 10.1016/j.tim.2004.08.008 . 15381196 PMC7119031

[9] Ravindra N, Alfajaro M, Gasque V, Huston N, Wan H, Szigeti-Buck K, et al. Single-cell longitudinal analysis of SARS-CoV-2 infection in human airway epithelium identifies target cells, alterations in gene expression, and cell state changes. PLoS Biol. 2021;19(3). doi: 10.1371/journal.pbio.3001143 . 33730024 PMC8007021

[10] Fiege J, Thiede J, Nanda H, Matchett W, Moore P, Montanari N, et al. Single cell resolution of SARS-CoV-2 tropism, antiviral responses, and susceptibility to therapies in primary human airway epithelium. PLoS Pathog. 2021;17(1). doi: 10.1371/journal.ppat.1009292 . 33507952 PMC7872261

[11] Li W, Moore MJ, Vasilieva N, Sui J, Wong SK, Berne MA, et al. Angiotensin-converting enzyme 2 is a functional receptor for the SARS coronavirus. Nature. 2003;426(6965):450–454. doi: 10.1038/nature02145 ; PubMed Central PMCID: PMC7095016. 14647384 PMC7095016

[12] Letko M, Marzi A, Munster V. Functional assessment of cell entry and receptor usage for SARS-CoV-2 and other lineage B betacoronaviruses. Nat Microbiol. 2020;5(4):562–569. doi: 10.1038/s41564-020-0688-y 32094589 PMC7095430

[13] Hoffmann M, Kleine-Weber H, Schroeder S, Krüger N, Herrler T, Erichsen S, et al. SARS-CoV-2 Cell Entry Depends on ACE2 and TMPRSS2 and Is Blocked by a Clinically Proven Protease Inhibitor. Cell. 2020;181(2). doi: 10.1016/j.cell.2020.02.052 . 32142651 PMC7102627

[14] Raj VS, Mou H, Smits SL, Dekkers DH, Müller MA, Dijkman R, et al. Dipeptidyl peptidase 4 is a functional receptor for the emerging human coronavirus-EMC. Nature. 2013;495(7440):251–254. doi: 10.1038/nature12005 ; PubMed Central PMCID: PMC7095326. 23486063 PMC7095326

[15] Qing E, Hantak M, Galpalli G, Gallagher T. Evaluating MERS-CoV Entry Pathways. Methods Mol Biol. 2020;2099:9–20. doi: 10.1007/978-1-0716-0211-9_2 . 31883084 PMC7121971

[16] Walls A, Park Y, Tortorici M, Wall A, McGuire A, Veesler D. Structure, Function, and Antigenicity of the SARS-CoV-2 Spike Glycoprotein. Cell. 2020;181(2). doi: 10.1016/j.cell.2020.02.058 . 32155444 PMC7102599

[17] Ou X, Liu Y, Lei X, Li P, Mi D, Ren L, et al. Characterization of spike glycoprotein of SARS-CoV-2 on virus entry and its immune cross-reactivity with SARS-CoV. Nat Commun. 2020;11(1). doi: 10.1038/s41467-020-15562-9 . 32221306 PMC7100515

[18] Zang R, Gomez Castro M, McCune B, Zeng Q, Rothlauf P, Sonnek N, et al. TMPRSS2 and TMPRSS4 promote SARS-CoV-2 infection of human small intestinal enterocytes. Sci Immunol. 2020;5(47). doi: 10.1126/sciimmunol.abc3582 . 32404436 PMC7285829

[19] Yan R, Zhang Y, Li Y, Xia L, Guo Y, Zhou Q. Structural basis for the recognition of SARS-CoV-2 by full-length human ACE2. Science (New York, NY). 2020;367(6485). doi: 10.1126/science.abb2762 . 32132184 PMC7164635

[20] Snijder E, van der Meer Y, Zevenhoven-Dobbe J, Onderwater J, van der Meulen J, Koerten H, et al. Ultrastructure and origin of membrane vesicles associated with the severe acute respiratory syndrome coronavirus replication complex. J Virol. 2006;80(12). doi: 10.1128/JVI.02501-05 . 16731931 PMC1472606

[21] Knoops K, Kikkert M, Worm S, Zevenhoven-Dobbe J, van der Mee Y, Koster A, et al. SARS-coronavirus replication is supported by a reticulovesicular network of modified endoplasmic reticulum. PLoS Biol. 2008;6(9). doi: 10.1371/journal.pbio.0060226 . 18798692 PMC2535663

[22] Stertz S, Reichelt M, Spiegel M, Kuri T, Martínez-Sobrido L, García-Sastre A, et al. The intracellular sites of early replication and budding of SARS-coronavirus. Virology. 2007;361(2). doi: 10.1016/j.virol.2006.11.027 . 17210170 PMC7103305

[23] Wei J, Alfajaro M, DeWeirdt P, Hanna R, Lu-Culligan W, Cai W, et al. Genome-wide CRISPR Screens Reveal Host Factors Critical for SARS-CoV-2 Infection. Cell. 2021;184(1). doi: 10.1016/j.cell.2020.10.028 . 33147444 PMC7574718

[24] Biering S, Sarnik S, Wang E, Zengel J, Leist S, Schäfer A, et al. Genome-wide bidirectional CRISPR screens identify mucins as host factors modulating SARS-CoV-2 infection. Nat Genet. 2022. doi: 10.1038/s41588-022-01131-x . 35879412 PMC9355872

[25] Laporte M, Raeymaekers V, Van Berwaer R, Vandeput J, Marchand-Casas I, Thibaut H, et al. The SARS-CoV-2 and other human coronavirus spike proteins are fine-tuned towards temperature and proteases of the human airways. PLoS Pathog. 2021;17(4). doi: 10.1371/journal.ppat.1009500 . 33886690 PMC8061995

[26] Tseng C, Tseng J, Perrone L, Worthy M, Popov V, Peters C. Apical entry and release of severe acute respiratory syndrome-associated coronavirus in polarized Calu-3 lung epithelial cells. J Virol. 2005;79(15). doi: 10.1128/JVI.79.15.9470-9479.2005 . 16014910 PMC1181546

[27] Ogando N, Dalebout T, Zevenhoven-Dobbe J, Limpens R, van der Meer Y, Caly L, et al. SARS-coronavirus-2 replication in Vero E6 cells: replication kinetics, rapid adaptation and cytopathology. J Gen Virol. 2020;101(9). doi: 10.1099/jgv.0.001453 . 32568027 PMC7654748

[28] Zhu Y, Feng F, Hu G, Wang Y, Yu Y, Zhu Y, et al. A genome-wide CRISPR screen identifies host factors that regulate SARS-CoV-2 entry. Nat Commun. 2021;12(1). doi: 10.1038/s41467-021-21213-4 . 33574281 PMC7878750

[29] Schneider W, Luna J, Hoffmann H, Sánchez-Rivera F, Leal A, Ashbrook A, et al. Genome-Scale Identification of SARS-CoV-2 and Pan-coronavirus Host Factor Networks. Cell. 2021;184(1). doi: 10.1016/j.cell.2020.12.006 . 33382968 PMC7796900

[30] Daniloski Z, Jordan T, Wessels H, Hoagland D, Kasela S, Legut M, et al. Identification of Required Host Factors for SARS-CoV-2 Infection in Human Cells. Cell. 2021;184(1). doi: 10.1016/j.cell.2020.10.030 . 33147445 PMC7584921

[31] Wang R, Simoneau C, Kulsuptrakul J, Bouhaddou M, Travisano K, Hayashi J, et al. Genetic Screens Identify Host Factors for SARS-CoV-2 and Common Cold Coronaviruses. Cell. 2021;184(1). doi: 10.1016/j.cell.2020.12.004 . 33333024 PMC7723770

[32] Kratzel A, Kelly J, V’kovski P, Portmann J, Brüggemann Y, Todt D, et al. A genome-wide CRISPR screen identifies interactors of the autophagy pathway as conserved coronavirus targets. PLoS Biol. 2021;19(12). doi: 10.1371/journal.pbio.3001490 . 34962926 PMC8741300

[33] Synowiec A, Jedrysik M, Branicki W, Klajmon A, Lei J, Owczarek K, et al. Identification of Cellular Factors Required for SARS-CoV-2 Replication. Cell. 2021;10(11). doi: 10.3390/cells10113159 . 34831382 PMC8622730

[34] Rebendenne A, Roy P, Bonaventure B, Chaves Valadão A, Desmarets L, Arnaud-Arnould M, et al. Bidirectional genome-wide CRISPR screens reveal host factors regulating SARS-CoV-2, MERS-CoV and seasonal HCoVs. Nat Genet. 2022. doi: 10.1038/s41588-022-01110-2 . 35879413 PMC11627114

[35] Grodzki M, Bluhm A, Schaefer M, Tagmount A, Russo M, Sobh A, et al. Genome-scale CRISPR screens identify host factors that promote human coronavirus infection. Genome Med. 2022;14(1). doi: 10.1186/s13073-022-01013-1 . 35086559 PMC8792531

[36] Tada T, Fan C, Chen J, Kaur R, Stapleford K, Gristick H, et al. An ACE2 Microbody Containing a Single Immunoglobulin Fc Domain Is a Potent Inhibitor of SARS-CoV-2. Cell Rep. 2020;33(12). doi: 10.1016/j.celrep.2020.108528 . 33326798 PMC7705358

[37] Brevini T, Maes M, Webb G, John B, Fuchs C, Buescher G, et al. FXR inhibition may protect from SARS-CoV-2 infection by reducing ACE2. Nature. 2023;615(7950). doi: 10.1038/s41586-022-05594-0 . 36470304 PMC9977684

[38] Oudit G, Wang K, Viveiros A, Kellner M, Penninger J. Angiotensin-converting enzyme 2-at the heart of the COVID-19 pandemic. Cell. 2023;186(5). doi: 10.1016/j.cell.2023.01.039 . 36787743 PMC9892333

[39] Wei J, Patil A, Collings C, Alfajaro M, Liang Y, Cai W, et al. Pharmacological disruption of mSWI/SNF complex activity restricts SARS-CoV-2 infection. Nat Genet. 2023;55(3). doi: 10.1038/s41588-023-01307-z ; PubMed Central PMCID: PMC10011139. 36894709 PMC10011139

[40] Martínez-Flores D, Zepeda-Cervantes J, Cruz-Reséndiz A, Aguirre-Sampieri S, Sampieri A, Vaca L. SARS-CoV-2 Vaccines Based on the Spike Glycoprotein and Implications of New Viral Variants. Front Immunol. 2021;12. doi: 10.3389/fimmu.2021.701501 . 34322129 PMC8311925

[41] Atas-Ozcan H, Brault V, Duchon A, Herault Y. Dyrk1a from Gene Function in Development and Physiology to Dosage Correction across Life Span in Down Syndrome. Genes. 2021;12(11). doi: 10.3390/genes12111833 . 34828439 PMC8624927

[42] Tejedor F. Dyrk1a. In: Choi S, editor. Encyclopedia of Signaling Molecules. Springer, New York, NY: SpringerLink; 2016.

[43] Becker W, Weber Y, Wetzel K, Eirmbter K, Tejedor F, Joost H. Sequence characteristics, subcellular localization, and substrate specificity of DYRK-related kinases, a novel family of dual specificity protein kinases. J Biol Chem. 1998;273(40). doi: 10.1074/jbc.273.40.25893 . 9748265

[44] Guard S, Poss Z, Ebmeier C, Pagratis M, Simpson H, Taatjes D, et al. The nuclear interactome of DYRK1A reveals a functional role in DNA damage repair. Sci Rep. 2019;9(1). doi: 10.1038/s41598-019-42990-5 . 31024071 PMC6483993

[45] Smith D, Stevens M, Sudanagunta S, Bronson R, Makhinson M, Watabe A, et al. Functional screening of 2 Mb of human chromosome 21q22.2 in transgenic mice implicates minibrain in learning defects associated with Down syndrome. Nat Genet. 1997;16(1). doi: 10.1038/ng0597-28 . 9140392

[46] Kimura R, Kamino K, Yamamoto M, Nuripa A, Kida T, Kazui H, et al. The DYRK1A gene, encoded in chromosome 21 Down syndrome critical region, bridges between beta-amyloid production and tau phosphorylation in Alzheimer disease. Hum Mol Genet. 2007;16(1). doi: 10.1093/hmg/ddl437 . 17135279

[47] Tejedor F, Hämmerle B. MNB/DYRK1A as a multiple regulator of neuronal development. FEBS J. 2011;278(2). doi: 10.1111/j.1742-4658.2010.07954.x . 21156027

[48] García-Cerro S, Martínez P, Vidal V, Corrales A, Flórez J, Vidal R, et al. Overexpression of Dyrk1A is implicated in several cognitive, electrophysiological and neuromorphological alterations found in a mouse model of Down syndrome. PLoS ONE. 2014;9(9). doi: 10.1371/journal.pone.0106572 . 25188425 PMC4154723

[49] Park J, Song W, Chung K. Function and regulation of Dyrk1A: towards understanding Down syndrome. Cell Mol Life Sci. 2009;66(20). doi: 10.1007/s00018-009-0123-2 . 19685005 PMC11115655

[50] Park J, Chung K. New Perspectives of Dyrk1A Role in Neurogenesis and Neuropathologic Features of Down Syndrome. Exp Neurobiol. 2013;22(4). doi: 10.5607/en.2013.22.4.244 . 24465139 PMC3897685

[51] Park J, Yang E, Yoon J, Chung K. Dyrk1A overexpression in immortalized hippocampal cells produces the neuropathological features of Down syndrome. Mol Cell Neurosci. 2007;36(2). doi: 10.1016/j.mcn.2007.07.007 . 17720532

[52] Laguna A, Barallobre M, Marchena M, Mateus C, Ramírez E, Martínez-Cue C, et al. Triplication of DYRK1A causes retinal structural and functional alterations in Down syndrome. Hum Mol Genet. 2013;22(14). doi: 10.1093/hmg/ddt125 . 23512985

[53] Ji J, Lee H, Argiropoulos B, Dorrani N, Mann J, Martinez-Agosto J, et al. DYRK1A haploinsufficiency causes a new recognizable syndrome with microcephaly, intellectual disability, speech impairment, and distinct facies. Eur J Hum Genet. 2015;23(11). doi: 10.1038/ejhg.2015.71 . 25944381 PMC4613469

[54] Clift A, Coupland C, Keogh R, Hemingway H, Hippisley-Cox J. COVID-19 Mortality Risk in Down Syndrome: Results From a Cohort Study Of 8 Million Adults. Ann Intern Med. 2020. doi: 10.7326/M20-4986 . 33085509 PMC7592804

[55] De Toma I, Dierssen M. Network analysis of Down syndrome and SARS-CoV-2 identifies risk and protective factors for COVID-19. Sci Rep. 2021;11(1). doi: 10.1038/s41598-021-81451-w . 33479353 PMC7820501

[56] Espinosa J. Down Syndrome and COVID-19: A Perfect Storm? Cell Rep Med. 2020;1(2). doi: 10.1016/j.xcrm.2020.100019 . 32501455 PMC7252041

[57] Malle L, Gao C, Hur C, Truong H, Bouvier N, Percha B, et al. Individuals with Down syndrome hospitalized with COVID-19 have more severe disease. Genet Med. 2020. doi: 10.1038/s41436-020-01004-w . 33060835 PMC7936948

[58] Illouz T, Biragyn A, Frenkel-Morgenstern M, Weissberg O, Gorohovski A, Merzon E, et al. Specific Susceptibility to COVID-19 in Adults with Down Syndrome. Neuromolecular Med. 2021. doi: 10.1007/s12017-021-08651-5 . 33660221 PMC7929736

[59] Hüls A, Costa A, Dierssen M, Baksh R, Bargagna S, Baumer N, et al. Medical vulnerability of individuals with Down syndrome to severe COVID-19-data from the Trisomy 21 Research Society and the UK ISARIC4C survey. EClinicalMedicine. 2021;33. doi: 10.1016/j.eclinm.2021.100769 . 33644721 PMC7897934

[60] Tejedor F, Zhu X, Kaltenbach E, Ackermann A, Baumann A, Canal I, et al. Minibrain: a new protein kinase family involved in postembryonic neurogenesis in Drosophila. Neuron. 1995;14(2). doi: 10.1016/0896-6273(95)90286-4 . 7857639

[61] Aranda S, Laguna A, de la Luna S. DYRK family of protein kinases: evolutionary relationships, biochemical properties, and functional roles. FASEB journal: official publication of the Federation of American Societies for Exp Biol. 2011;25(2). doi: 10.1096/fj.10-165837 . 21048044

[62] Salichs E, Ledda A, Mularoni L, Albà M, de la Luna S. Genome-wide analysis of histidine repeats reveals their role in the localization of human proteins to the nuclear speckles compartment. PLoS Genet. 2009;5(3). doi: 10.1371/journal.pgen.1000397 . 19266028 PMC2644819

[63] Lepagnol-Bestel A, Zvara A, Maussion G, Quignon F, Ngimbous B, Ramoz N, et al. DYRK1A interacts with the REST/NRSF-SWI/SNF chromatin remodelling complex to deregulate gene clusters involved in the neuronal phenotypic traits of Down syndrome. Hum Mol Genet. 2009;18(8). doi: 10.1093/hmg/ddp047 . 19218269

[64] Becker W, Sippl W. Activation, regulation, and inhibition of DYRK1A. FEBS J. 2011;278(2). doi: 10.1111/j.1742-4658.2010.07956.x . 21126318

[65] Alvarez M, Altafaj X, Aranda S, de la Luna S. DYRK1A autophosphorylation on serine residue 520 modulates its kinase activity via 14-3-3 binding. Mol Biol Cell. 2007;18(4). doi: 10.1091/mbc.e06-08-0668 . 17229891 PMC1838983

[66] Himpel S, Panzer P, Eirmbter K, Czajkowska H, Sayed M, Packman L, et al. Identification of the autophosphorylation sites and characterization of their effects in the protein kinase DYRK1 A. Biochem J. 2001;359(Pt 3). doi: 10.1042/0264-6021:3590497. . 11672423 PMC1222170

[67] Kentrup H, Becker W, Heukelbach J, Wilmes A, Schürmann A, Huppertz C, et al. Dyrk, a dual specificity protein kinase with unique structural features whose activity is dependent on tyrosine residues between subdomains VII and VIII. J Biol Chem. 1996;271(7). doi: 10.1074/jbc.271.7.3488 . 8631952

[68] Lochhead P, Sibbet G, Morrice N, Cleghon V. Activation-loop autophosphorylation is mediated by a novel transitional intermediate form of DYRKs. Cell. 2005;121(6). doi: 10.1016/j.cell.2005.03.034 . 15960979

[69] Sitz J, Tigges M, Baumgärtel K, Khaspekov L, Lutz B. Dyrk1A potentiates steroid hormone-induced transcription via the chromatin remodeling factor Arip4. Mol Cell Biol. 2004;24(13). doi: 10.1128/MCB.24.13.5821-5834.2004 . 15199138 PMC480880

[70] Di Vona C, Bezdan D, Islam A, Salichs E, López-Bigas N, Ossowski S, et al. Chromatin-wide profiling of DYRK1A reveals a role as a gene-specific RNA polymerase II CTD kinase. Mol Cell. 2015;57(3):506–20. Epub 2015/01/22. doi: 10.1016/j.molcel.2014.12.026 . 25620562

[71] Qian W, Jin N, Shi J, Yin X, Jin X, Wang S, et al. Dual-specificity tyrosine phosphorylation-regulated kinase 1A (Dyrk1A) enhances tau expression. J Alzheimers Dis. 2013;37(3). doi: 10.3233/JAD-130824 . 23948904

[72] von Groote-Bidlingmaier F, Schmoll D, Orth H, Joost H, Becker W, Barthel A. DYRK1 is a co-activator of FKHR (FOXO1a)-dependent glucose-6-phosphatase gene expression. Biochem Biophys Res Commun. 2003;300(3). doi: 10.1016/s0006-291x(02)02914-5 . 12507516

[73] Kelly P, Rahmani Z. DYRK1A enhances the mitogen-activated protein kinase cascade in PC12 cells by forming a complex with Ras, B-Raf, and MEK1. Mol Biol Cell. 2005;16(8). doi: 10.1091/mbc.e04-12-1085 . 15917294 PMC1182298

[74] Kung J, Jura N. Structural Basis for the Non-catalytic Functions of Protein Kinases. Structure. 2016; 24(1):7–24. doi: 10.1016/j.str.2015.10.020 . 26745528 PMC4706642

[75] Liang Y, Chang H, Wang C, Yu W. DYRK1A stabilizes HPV16E7 oncoprotein through phosphorylation of the threonine 5 and threonine 7 residues. Int J Biochem Cell Biol. 2008;40(11). doi: 10.1016/j.biocel.2008.04.003 . 18468476

[76] Cohen M, Yousef A, Massimi P, Fonseca G, Todorovic B, Pelka P, et al. Dissection of the C-terminal region of E1A redefines the roles of CtBP and other cellular targets in oncogenic transformation. J Virol. 2013;87(18). doi: 10.1128/JVI.00786-13 . 23864635 PMC3753994

[77] Hamilton S, Hutterer C, Egilmezer E, Steingruber M, Milbradt J, Marschall M, et al. Human cytomegalovirus utilises cellular dual-specificity tyrosine phosphorylation-regulated kinases during placental replication. Placenta. 2018;72–73:10–19. doi: 10.1016/j.placenta.2018.10.002 . 30501876

[78] Hutterer C, Milbradt J, Hamilton S, Zaja M, Leban J, Henry C, et al. Inhibitors of dual-specificity tyrosine phosphorylation-regulated kinases (DYRK) exert a strong anti-herpesviral activity. Antiviral Res. 2017;143. doi: 10.1016/j.antiviral.2017.04.003 . 28400201

[79] Kisaka J, Ratner L, Kyei G. The Dual-Specificity Kinase DYRK1A Modulates the Levels of Cyclin L2 To Control HIV Replication in Macrophages. J Virol. 2020;94(6). doi: 10.1128/JVI.01583-19 . 31852782 PMC7158737

[80] Booiman T, Loukachov V, van Dort K, van ’t Wout A, Kootstra N. DYRK1A Controls HIV-1 Replication at a Transcriptional Level in an NFAT Dependent Manner. PLoS ONE. 2015;10(12). doi: 10.1371/journal.pone.0144229 . 26641855 PMC4979971

[81] Xie X, Muruato A, Lokugamage K, Narayanan K, Zhang X, Zou J, et al. An Infectious cDNA Clone of SARS-CoV-2. Cell Host Microbe. 2020;27(5):841–848.e3. Epub 2020/04/13. doi: 10.1016/j.chom.2020.04.004 ; PubMed Central PMCID: PMC7153529. 32289263 PMC7153529

[82] Kii I, Sumida Y, Goto T, Sonamoto R, Okuno Y, Yoshida S, et al. Selective inhibition of the kinase DYRK1A by targeting its folding process. Nat Commun. 2016;7(1):1–14. doi: 10.1038/ncomms11391 27102360 PMC4844702

[83] Menon V, Ananthapadmanabhan V, Swanson S, Saini S, Sesay F, Yakovlev V, et al. DYRK1A regulates the recruitment of 53BP1 to the sites of DNA damage in part through interaction with RNF169. Cell Cycle. 2019;18(5). doi: 10.1080/15384101.2019.1577525 . 30773093 PMC6464593

[84] Ryoo S, Jeong H, Radnaabazar C, Yoo J, Cho H, Lee H, et al. DYRK1A-mediated hyperphosphorylation of Tau. A functional link between Down syndrome and Alzheimer disease. J Biol Chem. 2007;282(48). doi: 10.1074/jbc.M707358200 . 17906291

[85] Wiechmann S, Czajkowska H, de Graaf K, Grötzinger J, Joost H, Becker W. Unusual function of the activation loop in the protein kinase DYRK1A. Biochem Biophys Res Commun. 2003;302(2). doi: 10.1016/s0006-291x(03)00148-7 . 12604362

[86] Soppa U, Schumacher J, Florencio Ortiz V, Pasqualon T, Tejedor F, Becker W. The Down syndrome-related protein kinase DYRK1A phosphorylates p27(Kip1) and Cyclin D1 and induces cell cycle exit and neuronal differentiation. Cell Cycle. 2014;13(13). doi: 10.4161/cc.29104 . 24806449 PMC4111700

[87] Park J, Oh Y, Yoo L, Jung M, Song W, Lee S, et al. Dyrk1A phosphorylates p53 and inhibits proliferation of embryonic neuronal cells. J Biol Chem. 2010;285(41). doi: 10.1074/jbc.M110.147520 . 20696760 PMC2951261

[88] Yabut O, Domogauer J, D’Arcangelo G. Dyrk1A overexpression inhibits proliferation and induces premature neuronal differentiation of neural progenitor cells. The Journal of neuroscience: the official journal of the Society for Neuroscience. 2010;30(11). doi: 10.1523/JNEUROSCI.4711-09.2010 . 20237271 PMC3842457

[89] Branca C, Shaw D, Belfiore R, Gokhale V, Shaw A, Foley C, et al. Dyrk1 inhibition improves Alzheimer’s disease-like pathology. Aging Cell. 2017;16(5). doi: 10.1111/acel.12648 . 28779511 PMC5595697

[90] Göckler N, Jofre G, Papadopoulos C, Soppa U, Tejedor F, Becker W. Harmine specifically inhibits protein kinase DYRK1A and interferes with neurite formation. FEBS J. 2009;276(21). doi: 10.1111/j.1742-4658.2009.07346.x . 19796173

[91] Bain J, Plater L, Elliott M, Shpiro N, Hastie C, McLauchlan H, et al. The selectivity of protein kinase inhibitors: a further update. Biochem J. 2007;408(3). doi: 10.1042/BJ20070797 . 17850214 PMC2267365

[92] Ogawa Y, Nonaka Y, Goto T, Ohnishi E, Hiramatsu T, Kii I, et al. Development of a novel selective inhibitor of the Down syndrome-related kinase Dyrk1A. Nat Commun. 2010;1(1):1–9. doi: 10.1038/ncomms1090 20981014

[93] Rokey S, Foley C, Shaw Y, Bartholomew SW, W, Dunckley T, Velazquez R, et al., editors. Development of DYR533, a highly selective and orally bioavailable inhibitor of DYRK1A toward the treatment of Alzheimer’s disease and/or Down syndrome. 264th ACS National Meeting; 2022; Chicago, IL, USA.

[94] Travaglini K, Nabhan A, Penland L, Sinha R, Gillich A, Sit R, et al. A molecular cell atlas of the human lung from single-cell RNA sequencing. Nature. 2020;587(7835). doi: 10.1038/s41586-020-2922-4 . 33208946 PMC7704697

[95] Madissoon E, Wilbrey-Clark A, Miragaia R, Saeb-Parsy K, Mahbubani K, Georgakopoulos N, et al. scRNA-seq assessment of the human lung, spleen, and esophagus tissue stability after cold preservation. Genome Biol. 2019;21(1). doi: 10.1186/s13059-019-1906-x . 31892341 PMC6937944

[96] Mulay A, Konda B, Garcia G, Yao C, Beil S, Villalba J, et al. SARS-CoV-2 infection of primary human lung epithelium for COVID-19 modeling and drug discovery. Cell Rep. 2021;35(5). doi: 10.1016/j.celrep.2021.109055 . 33905739 PMC8043574

[97] Fotaki V, Dierssen M, Alcántara S, Martínez S, Martí E, Casas C, et al. Dyrk1A haploinsufficiency affects viability and causes developmental delay and abnormal brain morphology in mice. Mol Cell Biol. 2002;22(18). doi: 10.1128/MCB.22.18.6636-6647.2002 . 12192061 PMC135639

[98] Baggen J, Persoons L, Vanstreels E, Jansen S, Van Looveren D, Boeckx B, et al. Genome-wide CRISPR screening identifies TMEM106B as a proviral host factor for SARS-CoV-2. Nat Genet. 2021;53(4):435–444. doi: 10.1038/s41588-021-00805-2 33686287

[99] Thomas S, Moreira E, Kitchin N, Absalon J, Gurtman A, Lockhart S, et al. Safety and Efficacy of the BNT162b2 mRNA Covid-19 Vaccine through 6 Months. N Engl J Med. 2021;385(19). doi: 10.1056/NEJMoa2110345 . 34525277 PMC8461570

[100] Marovich M, Mascola J, Cohen M. Monoclonal Antibodies for Prevention and Treatment of COVID-19. JAMA. 2020;324(2). doi: 10.1001/jama.2020.10245 . 32539093

[101] Beigel J, Tomashek K, Dodd L, Mehta A, Zingman B, Kalil A, et al. Remdesivir for the Treatment of Covid-19—Final Report. N Engl J Med. 2020;383(19). doi: 10.1056/NEJMoa2007764 . 32445440 PMC7262788

[102] Hammond J, Leister-Tebbe H, Gardner A, Abreu P, Bao W, Wisemandle W, et al. Oral Nirmatrelvir for High-Risk, Nonhospitalized Adults with Covid-19. N Engl J Med. 2022;386(15). doi: 10.1056/NEJMoa2118542 . 35172054 PMC8908851

[103] Mahase E. Covid-19: Molnupiravir reduces risk of hospital admission or death by 50% in patients at risk, MSD reports. BMJ. 2021;375. doi: 10.1136/bmj.n2422 . 34607801

[104] Zumla A, Hui D, Azhar E, Memish Z, Maeurer M. Reducing mortality from 2019-nCoV: host-directed therapies should be an option. Lancet. (London, England). 2020;395(10224). doi: 10.1016/S0140-6736(20)30305-6 . 32035018 PMC7133595

[105] Plante J, Mitchell B, Plante K, Debbink K, Weaver S, Menachery V. The variant gambit: COVID-19’s next move. Cell Host Microbe. 2021;29(4). doi: 10.1016/j.chom.2021.02.020 . 33789086 PMC7919536

[106] Shehata H, Tarpley M, Oladapo H, Strepay D, Roques J, Onyenwoke R, et al. Profiling of harmine and select analogs as differential inhibitors of DYRK1A and monoamine oxidase A: Exploring the potential for anti-cancer efficacy and minimizing off-target activity | Molecular Cancer Therapeutics | American Association for Cancer Research. Mol Cancer Ther. 2022;18. doi: 10.1158/1535-7163.TARG-19-A138

[107] Jarhad D, Mashelkar K, Kim H, Noh M, Jeong L. Dual-Specificity Tyrosine Phosphorylation-Regulated Kinase 1A (DYRK1A) Inhibitors as Potential Therapeutics. J Med Chem. 2018;61(22):9791–810. Epub 2018/07/20. doi: 10.1021/acs.jmedchem.8b00185 . 29985601

[108] Li X, Pu W, Zheng Q, Ai M, Chen S, Peng Y. Proteolysis-targeting chimeras (PROTACs) in cancer therapy. Mol Cancer. 2022;21(1). doi: 10.1186/s12943-021-01434-3 . 35410300 PMC8996410

[109] Desantis J, Goracci L. Proteolysis targeting chimeras in antiviral research. Future Med Chem. 2022;14(7). doi: 10.4155/fmc-2022-0005 . 35134309

[110] Sonamoto R, Kii I, Koike Y, Sumida Y, Kato-Sumida T, Okuno Y, et al. Identification of a DYRK1A Inhibitor that Induces Degradation of the Target Kinase using Co-chaperone CDC37 fused with Luciferase nanoKAZ. Sci Rep. 2015;5(1):1–13. doi: 10.1038/srep12728 26234946 PMC4522657

[111] Goswami R, Russell V, Tu J, Thomas C, Hughes P, Kelly F, et al. Oral Hsp90 inhibitor SNX-5422 attenuates SARS-CoV-2 replication and dampens inflammation in airway cells. iScience. 2021;24(12). doi: 10.1016/j.isci.2021.103412 . 34786537 PMC8579697

[112] Li S, Xu C, Fu Y, Lei P, Yao Y, Yang W, et al. DYRK1A interacts with histone acetyl transferase p300 and CBP and localizes to enhancers. Nucleic Acids Res. 2018;46(21). doi: 10.1093/nar/gky754 . 30137413 PMC6265467

[113] Jang S, Azebi S, Soubigou G, Muchardt C. DYRK1A phoshorylates histone H3 to differentially regulate the binding of HP1 isoforms and antagonize HP1-mediated transcriptional repression. EMBO Rep. 2014;15(6). doi: 10.15252/embr.201338356 . 24820035 PMC4197879

[114] Kinstrie R, Lochhead P, Sibbet G, Morrice N, Cleghon V. dDYRK2 and Minibrain interact with the chromatin remodelling factors SNR1 and TRX. Biochem J. 2006;398(1). doi: 10.1042/BJ20060159 . 16671894 PMC1525014

[115] Sherman E, Mirabelli C, Tang V, Khan T, Leix K, Kennedy A, et al. Identification of cell type specific ACE2 modifiers by CRISPR screening. PLoS Pathog. 2022;18(3). doi: 10.1371/journal.ppat.1010377 . 35231079 PMC8929698

[116] Wang X, Lee R, Alver B, Haswell J, Wang S, Mieczkowski J, et al. SMARCB1-mediated SWI/SNF complex function is essential for enhancer regulation. Nat Genet. 2017;49(2). doi: 10.1038/ng.3746 . 27941797 PMC5285474

[117] Imbalzano A, Imbalzano K, Nickerson J. BRG1, a SWI/SNF chromatin remodeling enzyme ATPase, is required for maintenance of nuclear shape and integrity. Commun Integr Biol. 2013;6(5). doi: 10.4161/cib.25153 . 24228137 PMC3821668

[118] Froimchuk E, Jang Y, Ge K. Histone H3 lysine 4 methyltransferase KMT2 D. Gene. 2017;627. doi: 10.1016/j.gene.2017.06.056 . 28669924 PMC5546304

[119] Beacon T, Delcuve G, Davie J. Epigenetic regulation of ACE2, the receptor of the SARS-CoV-2 virus 1. Genome. 2021;64(4). doi: 10.1139/gen-2020-0124 . 33086021

[120] Pedersen K, Chhabra K, Nguyen V, Xia H, Lazartigues E. The transcription factor HNF1α induces expression of angiotensin-converting enzyme 2 (ACE2) in pancreatic islets from evolutionarily conserved promoter motifs. Biochim Biophys Acta. 2013;1829(11). doi: 10.1016/j.bbagrm.2013.09.007 . 24100303 PMC3838857

[121] Israeli M, Finkel Y, Yahalom-Ronen Y, Paran N, Chitlaru T, Israeli O, et al. Genome-wide CRISPR screens identify GATA6 as a proviral host factor for SARS-CoV-2 via modulation of ACE2. Nat Commun. 2022;13(1). doi: 10.1038/s41467-022-29896-z . 35469023 PMC9039069

[122] Röhrborn D, Wronkowitz N, Eckel J. DPP4 in Diabetes. Front Immunol. 2015;6. doi: 10.3389/fimmu.2015.00386 . 26284071 PMC4515598

[123] Mulvihill E, Drucker D. Pharmacology, physiology, and mechanisms of action of dipeptidyl peptidase-4 inhibitors. Endocr Rev. 2014;35(6). doi: 10.1210/er.2014-1035 . 25216328 PMC7108477

[124] Senkel S, Lucas B, Klein-Hitpass L, Ryffel G. Identification of target genes of the transcription factor HNF1beta and HNF1alpha in a human embryonic kidney cell line. Biochim Biophys Acta. 2005;1731(3). doi: 10.1016/j.bbaexp.2005.10.003 . 16297991

[125] Böhm S, Gum J, Erickson R, Hicks J, Kim Y. Human dipeptidyl peptidase IV gene promoter: tissue-specific regulation from a TATA-less GC-rich sequence characteristic of a housekeeping gene promoter. Biochem J. 1995;311 (Pt 3)(Pt 3). doi: 10.1042/bj3110835 . 7487939 PMC1136077

[126] Erickson R, Gum J, Lotterman C, Hicks J, Lai R, Kim Y. Regulation of the gene for human dipeptidyl peptidase IV by hepatocyte nuclear factor 1 alpha. Biochem J. 1999;338(Pt 1). . 9931303 PMC1220029

[127] Avanzato V, Oguntuyo K, Escalera-Zamudio M, Gutierrez B, Golden M, Kosakovsky Pond S, et al. A structural basis for antibody-mediated neutralization of Nipah virus reveals a site of vulnerability at the fusion glycoprotein apex. Proc Natl Acad Sci U S A. 2019;116(50). doi: 10.1073/pnas.1912503116 . 31767754 PMC6911215

[128] Lefrancois L, Lyles D. The interaction of antibody with the major surface glycoprotein of vesicular stomatitis virus. I. Analysis of neutralizing epitopes with monoclonal antibodies. Virology. 1982;121(1). . 18638751

[129] Huang I, Bosch B, Li F, Li W, Lee K, Ghiran S, et al. SARS coronavirus, but not human coronavirus NL63, utilizes cathepsin L to infect ACE2-expressing cells. J Biol Chem. 2006;281(6). doi: 10.1074/jbc.M508381200 . 16339146 PMC8010168

[130] Dobin A, Davis C, Schlesinger F, Drenkow J, Zaleski C, Jha S, et al. STAR: ultrafast universal RNA-seq aligner. Bioinformatics. 2013;29(1). doi: 10.1093/bioinformatics/bts635 . 23104886 PMC3530905

[131] Love M, Huber W, Anders S. Moderated estimation of fold change and dispersion for RNA-seq data with DESeq2. Genome Biol. 2014;15(12). doi: 10.1186/s13059-014-0550-8 . 25516281 PMC4302049

[132] Gu Z, Eils R, Schlesner M. Complex heatmaps reveal patterns and correlations in multidimensional genomic data. Bioinformatics. 2016;32(18). doi: 10.1093/bioinformatics/btw313 . 27207943

[133] Bolger A, Lohse M, Usadel B. Trimmomatic: a flexible trimmer for Illumina sequence data. Bioinformatics. 2014;30(15). doi: 10.1093/bioinformatics/btu170 . 24695404 PMC4103590

[134] Langmead B, Salzberg S. Fast gapped-read alignment with Bowtie 2. Nat Methods. 2012;9(4). doi: 10.1038/nmeth.1923 . 22388286 PMC3322381

[135] Li H, Handsaker B, Wysoker A, Fennell T, Ruan J, Homer N, et al. The Sequence Alignment/Map format and SAMtools. Bioinformatics. 2009;25(16). doi: 10.1093/bioinformatics/btp352 . 19505943 PMC2723002

[136] Zhang Y, Liu T, Meyer C, Eeckhoute J, Johnson D, Bernstein B, et al. Model-based analysis of ChIP-Seq (MACS). Genome Biol. 2008;9(9). doi: 10.1186/gb-2008-9-9-r137 . 18798982 PMC2592715

[137] Liao Y, Smyth G, Shi W. featureCounts: an efficient general purpose program for assigning sequence reads to genomic features. Bioinformatics. 2014;30(7). doi: 10.1093/bioinformatics/btt656 . 24227677

[138] Ramírez F, Ryan D, Grüning B, Bhardwaj V, Kilpert F, Richter A, et al. deepTools2: a next generation web server for deep-sequencing data analysis. Nucleic Acids Res. 2016;44(W1). doi: 10.1093/nar/gkw257 . 27079975 PMC4987876

[139] Thompson B, Bhansali R, Diebold L, Cook D, Stolzenburg L, Casagrande A, et al. DYRK1A controls the transition from proliferation to quiescence during lymphoid development by destabilizing Cyclin D3. J Exp Med. 2015;212(6). doi: 10.1084/jem.20150002 . 26008897 PMC4451127

[140] Ruzankina Y, Pinzon-Guzman C, Asare A, Ong T, Pontano L, Cotsarelis G, et al. Deletion of the developmentally essential gene ATR in adult mice leads to age-related phenotypes and stem cell loss. Cell Stem Cell. 2007;1(1). doi: 10.1016/j.stem.2007.03.002 . 18371340 PMC2920603

[141] Hao Y, Hao S, Andersen-Nissen E, Mauck W, Zheng S, Butler A, et al. Integrated analysis of multimodal single-cell data. Cell. 2021;184(13). doi: 10.1016/j.cell.2021.04.048 . 34062119 PMC8238499

