# Supplementary material for: DYRK1A promotes viral entry of highly pathogenic human coronaviruses in a kinase-independent manner
Source: PLoS Biol. 2023 Jun 13;21(6):e3002097. doi: 10.1371/journal.pbio.3002097 (PMC10263356; doi:10.1371/journal.pbio.3002097)
Supplement: S1 Raw Images — (PDF) [file pbio.3002097.s002.pdf]

Raw blot for generating Figure 1B

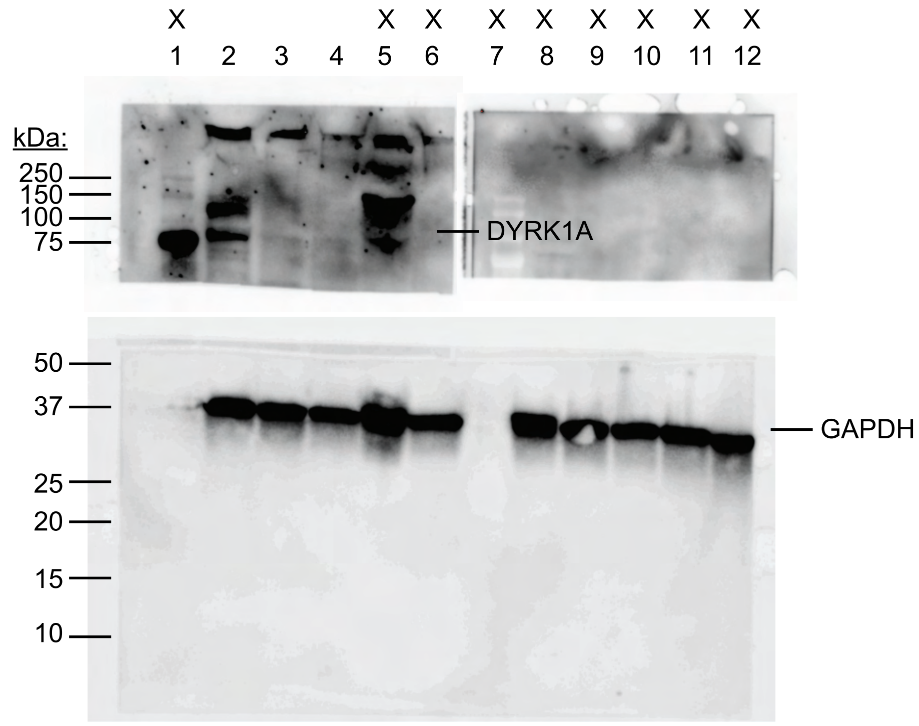

1. Ladder
2. WT Vero-E6
3. DYRK1A KO#1
4. DYRK1A KO#2
5. WT Huh7.5
6. H2_4 Clone DYRK1A putative heterozygote or incomplete KO
7. Ladder
8. WT Vero-E6
9. DYRK1A KO#1
10. DYRK1A KO#2
11. WT Huh7.5
12. H2_4 Clone DYRK1A putative heterozygote or incomplete KO

Lanes 2-5, 8-12: approx. 340k cells/well loaded
Samples were ran on the gel together, then the membrane was cut for antibody probing and imaging

α-DYRK1A blots (top) were stained with the same antibody, but top right was an attempt to reprobe again after stripping (antibody sensitivity doesn't work well in this context)

Imaged using auto-exposure (<1 min) on BioRad ChemiDoc Imaging System

Raw blot for generating Figure 2A

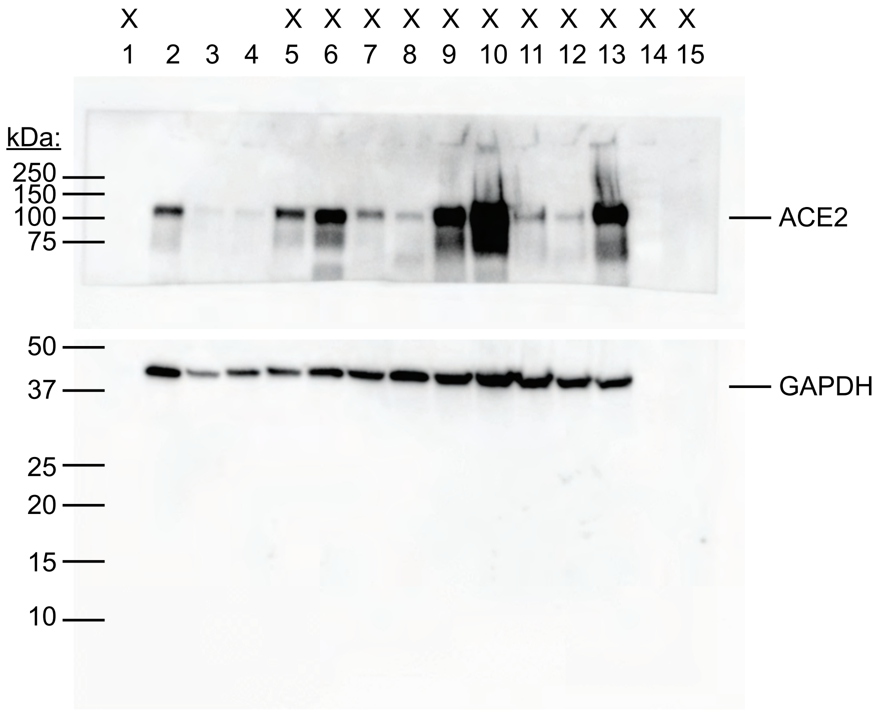

1. Ladder
2. WT Vero-E6
3. DYRK1A KO#1
4. DYRK1A KO#2
5. H2_4 Clone DYRK1A putative heterozygote
6. WT Vero-E6
7. DYRK1A KO#1
8. DYRK1A KO#2
9. H2_4 Clone DYRK1A putative heterozygote
10. WT Vero-E6
11. DYRK1A KO#1
12. DYRK1A KO#2
13. H2_4 Clone DYRK1A putative heterozygote
14. Ladder
15. Empty

Lanes 2-5: approx. 94k cells/well loaded
Lanes 6-9: approx. 188k cells/well loaded
Lanes 10-13: approx 282k cells/well loaded

Samples were ran on the gel together, then the membrane was cut for antibody probing and imaging

Lanes 2-5, 6-9, and 10-13 were all generated from independent splits (e.g., biological replicates) of each cell line

Imaged using auto-exposure (<1 min) on BioRad ChemiDoc Imaging System

Raw blot for generating Figure 2C

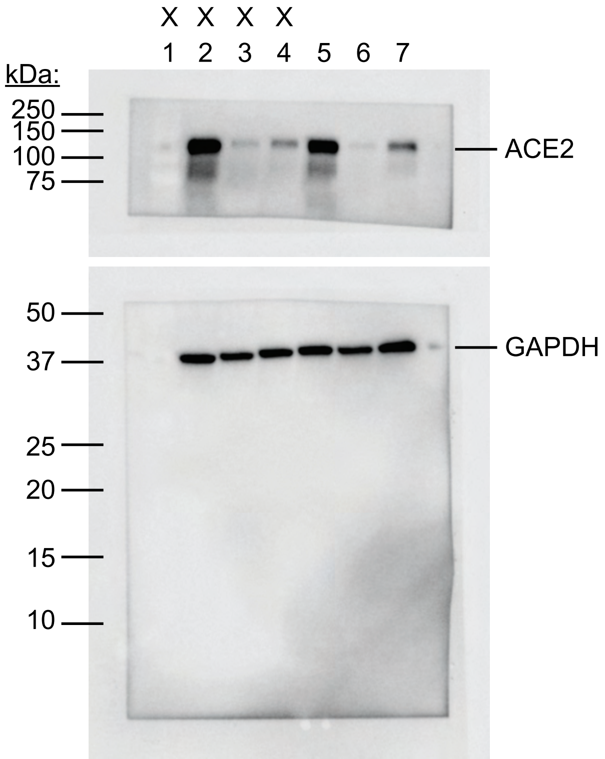

1. Ladder
2. WT Vero-E6
3. DYRK1A KO#1
4. DYRK1A KO#1 + hACE2
5. WT Vero-E6
6. DYRK1A KO#1
7. DYRK1A KO#1 + hACE2

Lanes 2-7: approx. 192k cells/well loaded

Lanes 2-4 and 5-7 were all generated from independent splits (e.g., biological replicates) of each cell line

Samples were ran on the gel together, then the membrane was cut for antibody probing and imaging

Imaged using auto-exposure (<1 min) on BioRad ChemiDoc Imaging System

Raw blot for generating Figure 2D

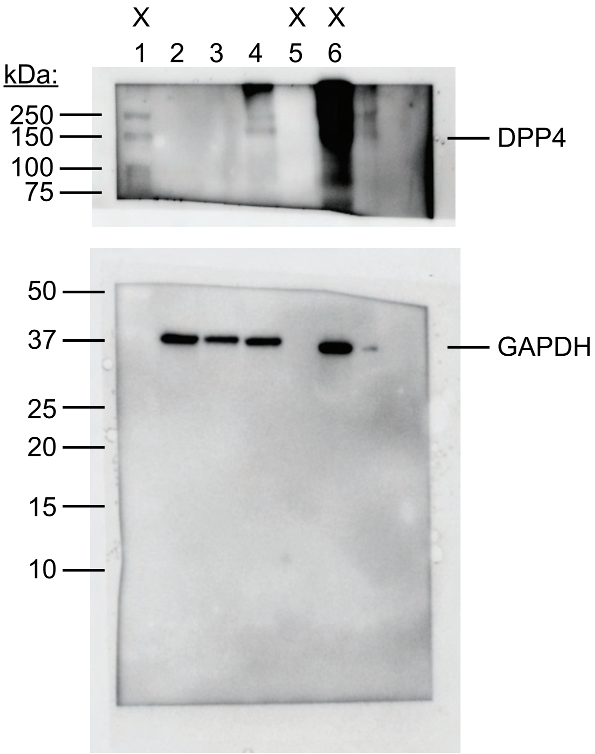

1. Ladder
2. WT Vero-E6
3. DYRK1A KO#2
4. DYRK1A KO#2 + hDPP4
5. Empty
6. 293T + hDPP4 strongly overexpressing positive control
7. Blank

Lanes 2-5, 6: approx. 192k cells/well loaded

Samples were ran on the gel together, then the membrane was cut for antibody probing and imaging

Imaged using auto-exposure (~3 min) on BioRad ChemiDoc Imaging System

Raw blot for generating Figure 3B

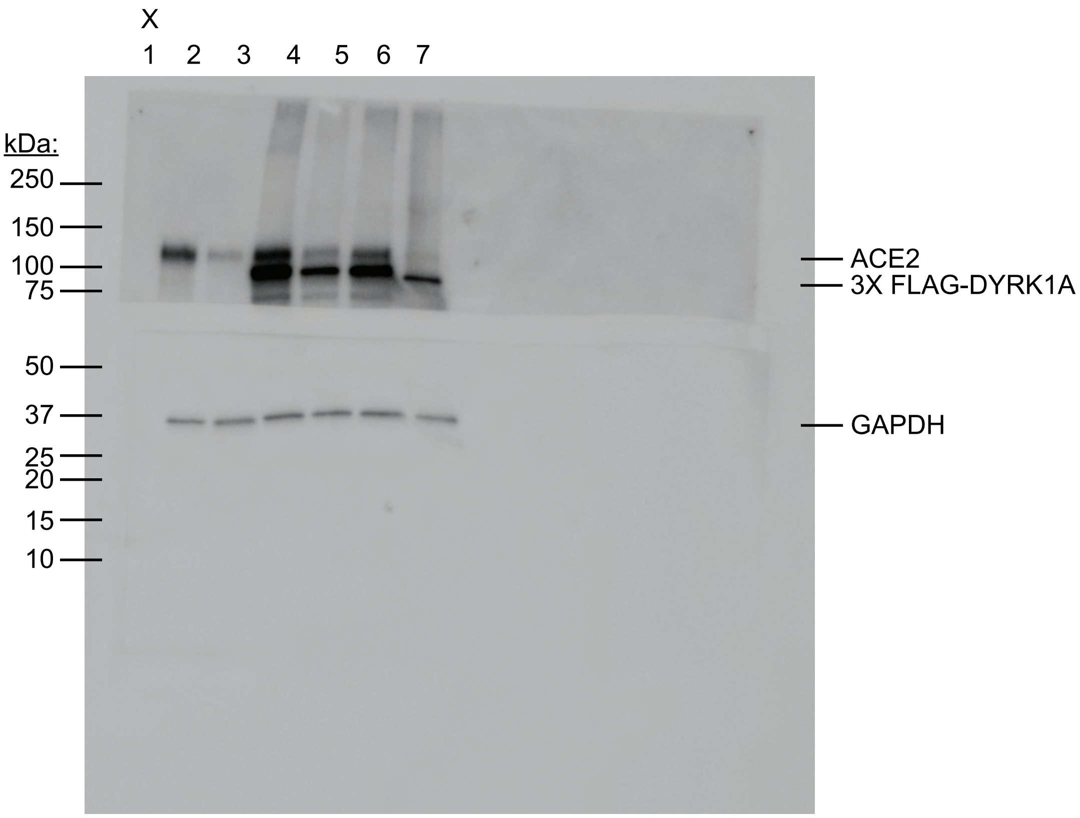

1. Ladder
2. WT Vero-E6
3. DYRK1A KO#1 + empty vector
4. DYRK1A KO#1 + WT 3X FLAG-DYRK1A
5. DYRK1A KO#1 + K188R 3X FLAG-DYRK1A
6. DYRK1A KO#1 + Y321F 3X FLAG-DYRK1A
7. DYRK1A KO#1 + NES 3X FLAG-DYRK1A

Remaining lanes - empty

Lanes 2-7: approx. 192k cells/well loaded

Samples were ran on the gel together, then the membrane was cut for antibody probing and imaging

Imaged using auto-exposure (~3 min) on BioRad ChemiDoc Imaging System

Raw blot for generating Figure 3C

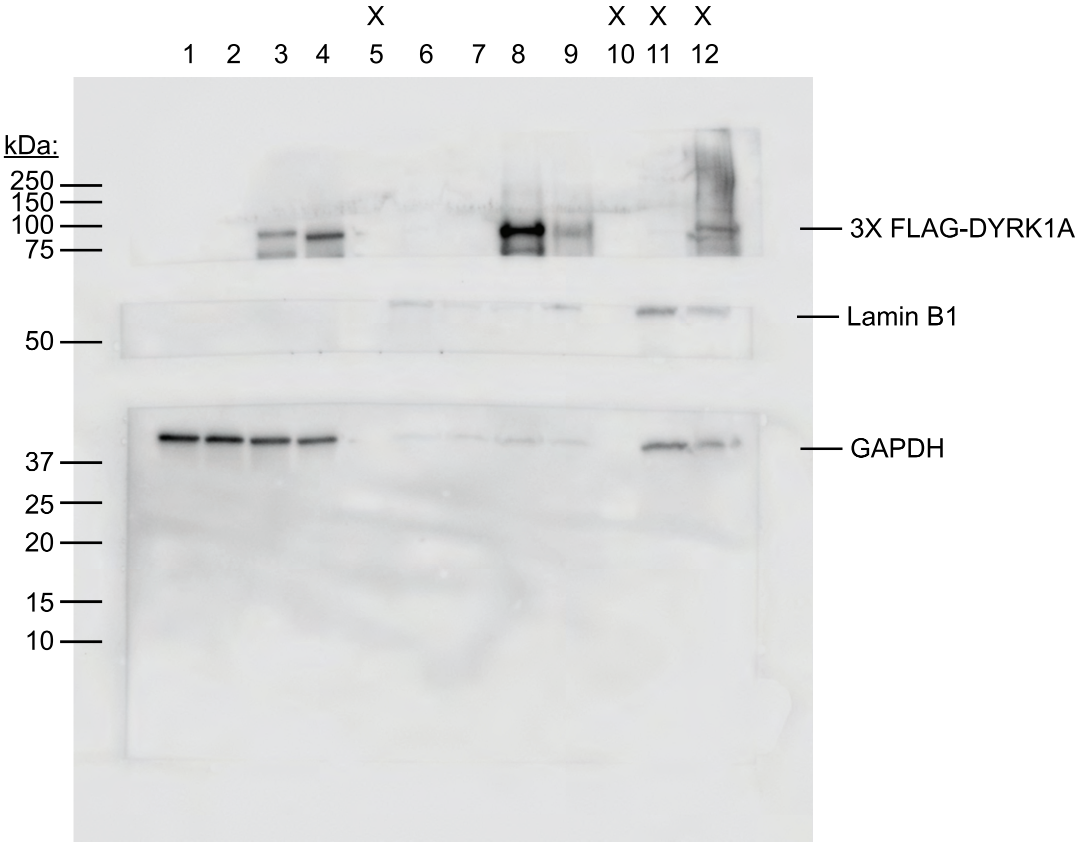

1. DYRK1A KO#1 + WT 3X FLAG-DYRK1A (no DOX) - cytosolic fraction
2. DYRK1A KO#1 + NES 3X FLAG-DYRK1A (no DOX) - cytosolic fraction
3. DYRK1A KO#1 + WT 3X FLAG-DYRK1A (+DOX) - cytosolic fraction
4. DYRK1A KO#1 + NES 3X FLAG-DYRK1A (+DOX) - cytosolic fraction
5. Ladder
6. DYRK1A KO#1 + WT 3X FLAG-DYRK1A (no DOX)
7. DYRK1A KO#1 + NES 3X FLAG-DYRK1A (no DOX)
8. DYRK1A KO#1 + WT 3X FLAG-DYRK1A (+DOX)
9. DYRK1A KO#1 + NES 3X FLAG-DYRK1A (+DOX)
10. Ladder
11. DYRK1A KO#1 + empty vector whole cell lysate (unfractionated, +DOX)
12. DYRK1A KO#1 + WT 3X FLAG-DYRK1A whole cell lysate (unfractionated, +DOX)

Approx. 1e6 cells were used for cysotolic/nuclear fractionation, and 12.5 µl/well were loaded after fractionation

Samples were ran on the gel together, then the membrane was cut for antibody probing and imaging

Imaged using auto-exposure (~3 min) on BioRad ChemiDoc Imaging System

Raw blot images for generating Figure 1C

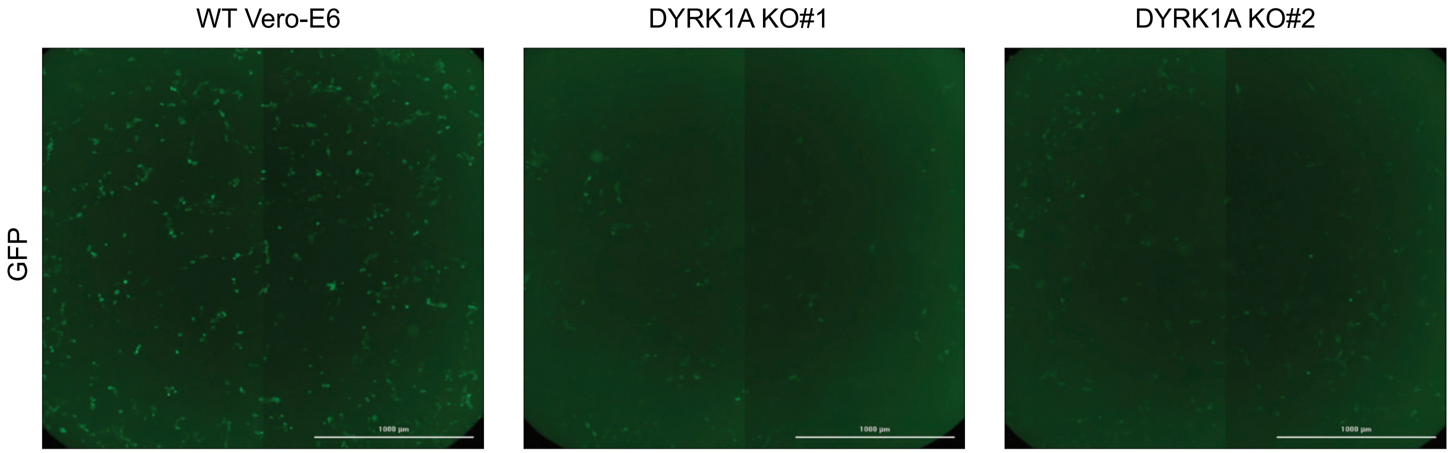

Imaged on Cytation 5 configured with Bright Field and GFP cubes with 4 stitched images/well
