# Supplementary figures and images for: DYRK1A promotes viral entry of highly pathogenic human coronaviruses in a kinase-independent manner
Source: PLoS Biol. 2023 Jun 13;21(6):e3002097. doi: 10.1371/journal.pbio.3002097 (PMC10263356; doi:10.1371/journal.pbio.3002097)

Supplemental Figure 1

VSVpp-Rluc-SARS2-S

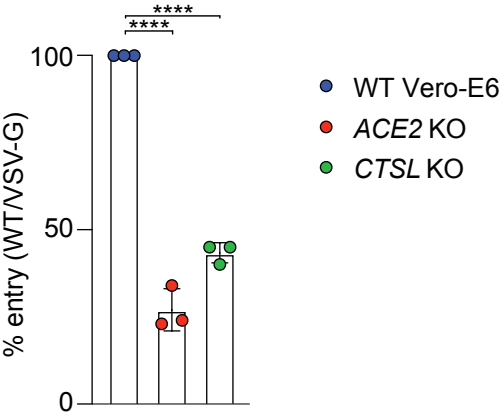

Supplement: S1 Fig — Polyclonal deletion of ACE2 or CTSL significantly reduce pseudotyped particles expressing the SARS-CoV-2 spike relative to WT Vero-E6 cells, like loss of DYRK1A. Cells were infected with VSVpp encoding CoV spike proteins and an Rluc reporter at 24 hpi. % Entry for VSVpp-Rluc-SARS2-S was normalized to VSVpp-Rluc-VSV-G control and WT Vero-E6 cells. Data were analyzed by unpaired Student t test; **** p < 0.0001. Shown are means ± SEM. Data are representative of 3 independent biological experiments performed with 3 technical replicates. Data underlying this figure can be found in S1 Data. ACE2, angiotensin-converting enzyme 2; CTSL, Cathepsin L; DYRK1A, Dual Specificity Tyrosine Phosphorylation Regulated Kinase 1A; hpi, hours postinfection; Rluc, Renilla luciferase; SARS-CoV-2, Severe Acute Respiratory Syndrome Coronavirus 2; VSVpp, VSV pseudovirus; WT, wild-type. (PDF) [file pbio.3002097.s003.pdf]

Supplemental Figure 2

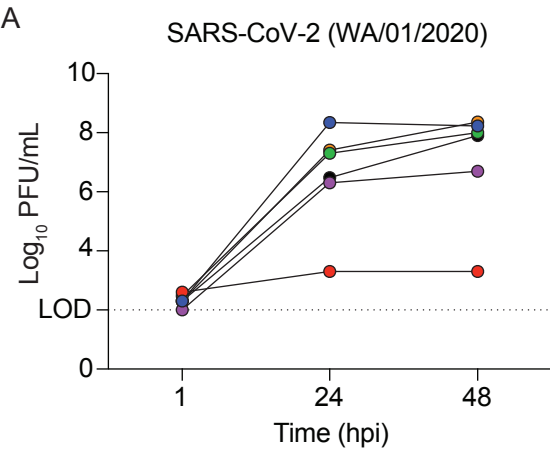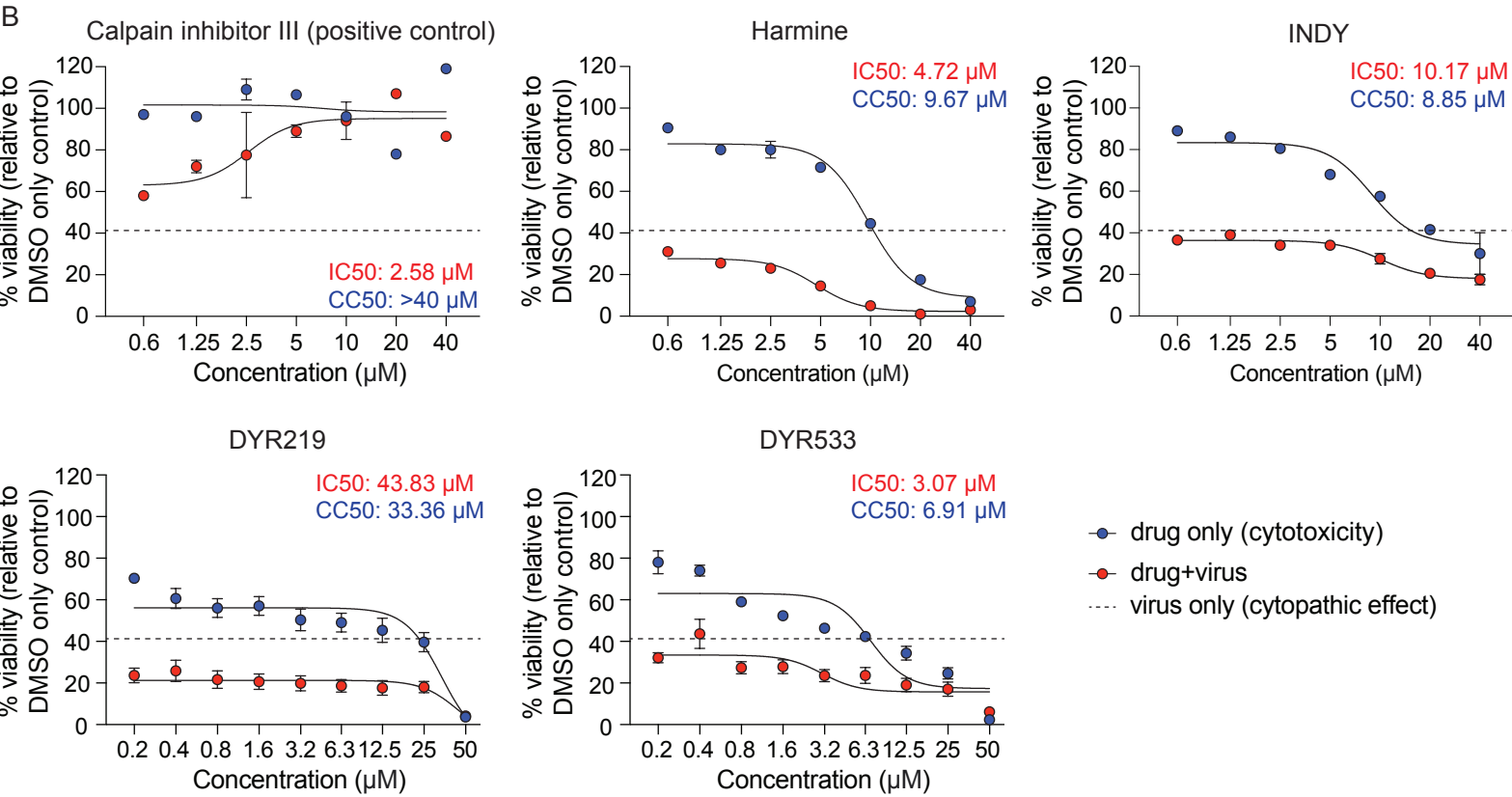

Supplement: S2 Fig — (A) WT Vero-E6 cells, DYRK1A KO cells, and cells overexpressing DYRK1A after DOX induction for 72 hours were infected with SARS-CoV-2 WA/01/2020 at an MOI of approximately 0.1 for 1, 24, or 48 hours. Plaque assays were performed in biological duplicate. Shown is 1 representative replicate. (B) WT Vero-E6 cells were treated with the positive control protease inhibitor calpain inhibitor III or a potent DYRK1A inhibitor (harmine, INDY, DYR219, and DYR533) for 48 hours. Cells were then infected with SARS-CoV-2 (MOI approximately 1), and cell viability was assessed 72 hpi via CellTiter-Glo. % Viability was calculated relative to uninfected or untreated controls. The half-maximal inhibitory concentration (IC50) and half-maximal cytotoxic concentration (CC50) were calculated for each drug using nonlinear regression dose response curves. Each experiment was performed at least 2 independent times. Shown are the means of 2–3 technical replicates. Data underlying this figure can be found in S1 Data. DOX, doxycycline; DYRK1A, Dual Specificity Tyrosine Phosphorylation Regulated Kinase 1A; hpi, hours postinfection; KO, knockout; SARS-CoV-2, Severe Acute Respiratory Syndrome Coronavirus 2; WT, wild-type. (PDF) [file pbio.3002097.s004.pdf]

Supplemental Figure 3

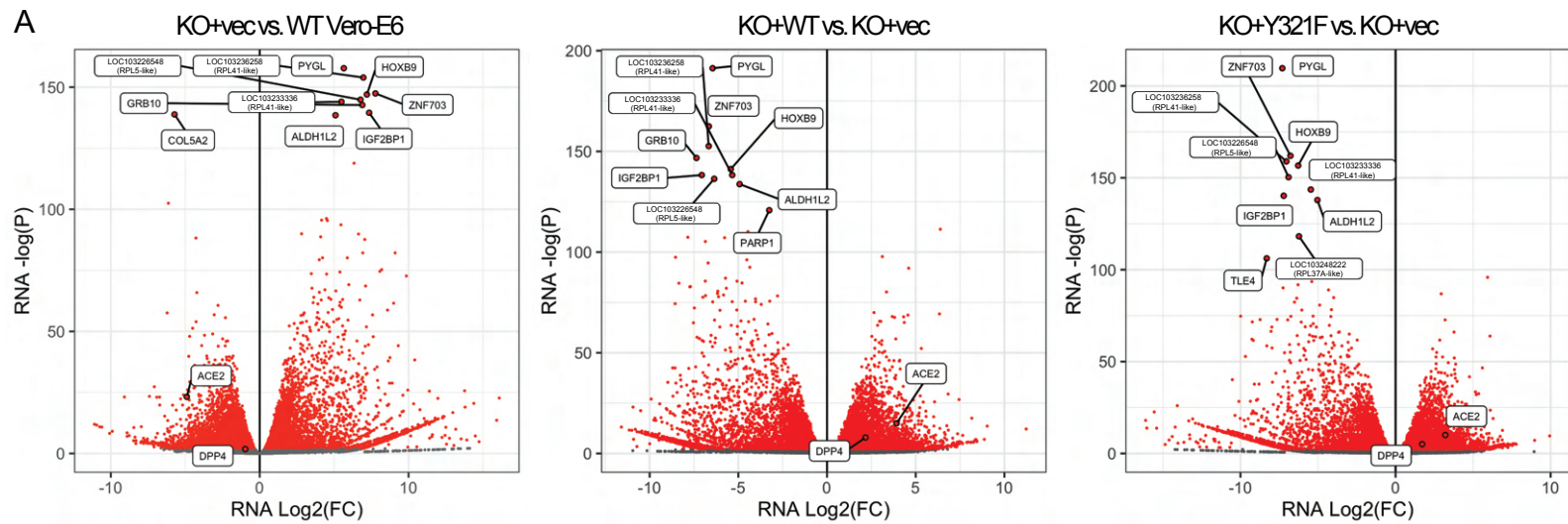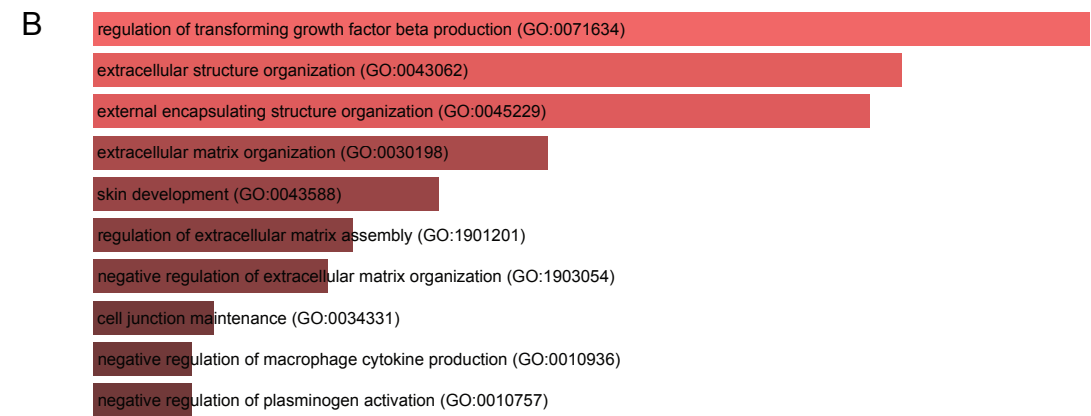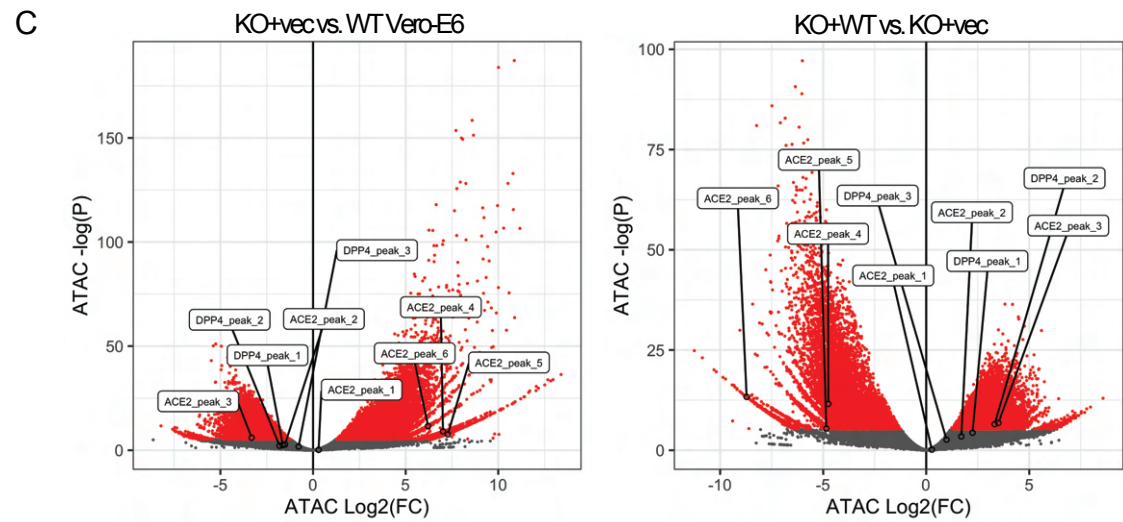

Supplement: S3 Fig — (A) RNA-Seq volcano plots depicting DEGs in cells where DYRK1A is absent or reintroduced. (B) Enrichr pathway analysis for biological function enriched in the presence of DYRK1A. Gene set criteria included p < 0.05, L2FC<0 for KO+vec vs. WT Vero-E6 and L2FC>1.5 for both KO+WT and KO+Y321F vs. KO+vec. (C) ATAC-Seq volcano plots depicting DEGs in cells where DYRK1A is absent or reintroduced. All experiments were performed in biological duplicate. Data underlying this figure can be found under GEO Accession GSE213999. DEG, differentially expressed gene; DYRK1A, Dual Specificity Tyrosine Phosphorylation Regulated Kinase 1A; KO, knockout; L2FC, log2 fold-change; WT, wild-type. (PDF) [file pbio.3002097.s005.pdf]

Supplemental Figure 4

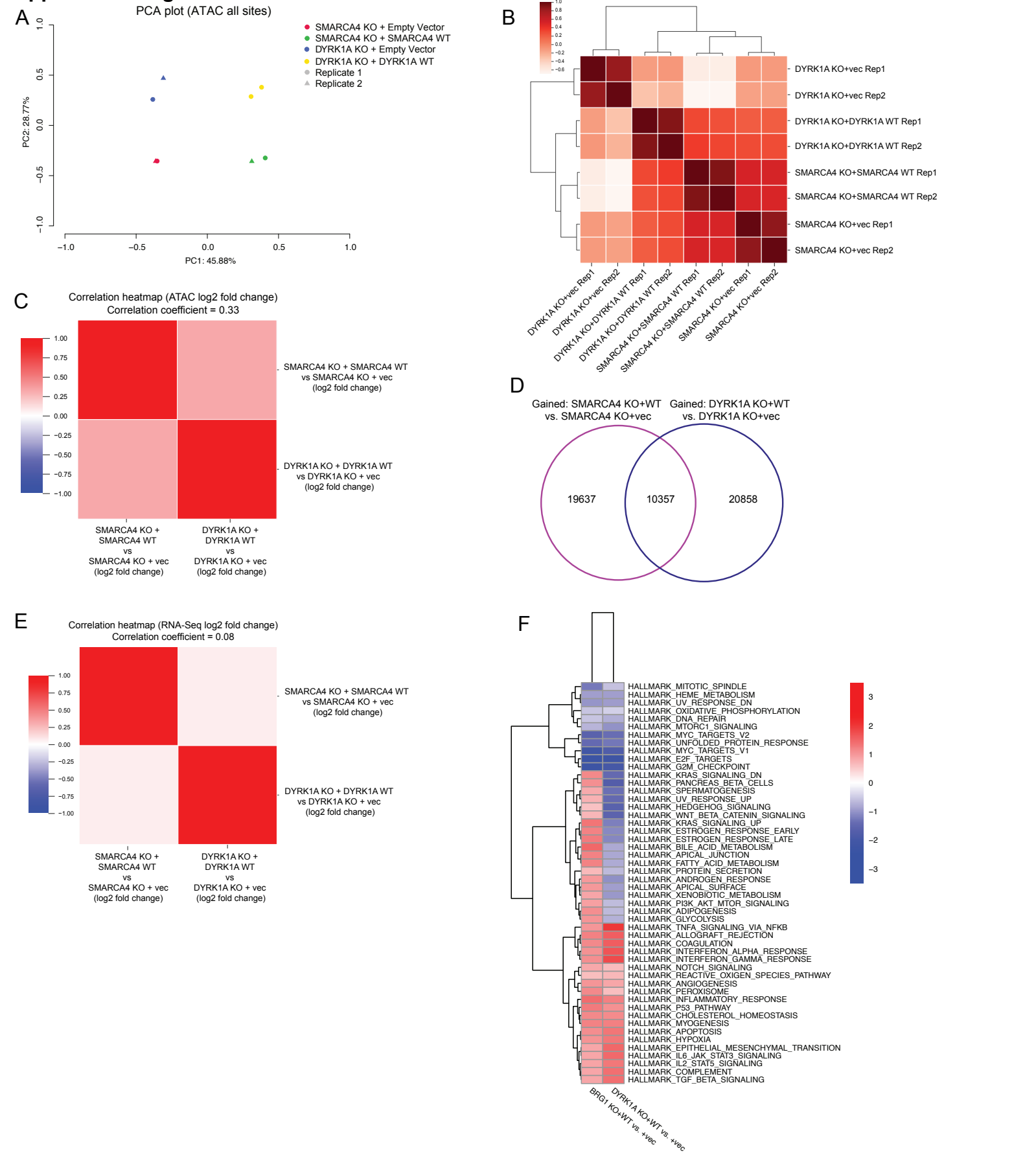

Supplement: S4 Fig — (A) Principal component analysis of ATAC-Seq experiments performed in DYRK1A KO+vec, DYRK1A KO+WT, SMARCA4 KO+vec, and SMARCA4 KO+WT cells generated in a parental Vero-E6 background. Each experiment was performed in biological duplicate (replicates 1 and 2). DYRK1A and SMARCA4 loss share some molecular impacts, suggesting that some pathways may be coregulated (PC1), whereas others may be independently regulated (PC2). (B) Correlation heatmap comparing all sites from ATAC-Seq experiments in DYRK1A KO+vec, DYRK1A KO+WT, SMARCA4 KO+vec, and SMARCA4 KO+WT cells. (C) Correlation heatmap comparing chromatin accessibility by ATAC-Seq in DYRK1A or SMARCA4 complemented cells, identifying a correlation coefficient of 0.33 supporting approximately 33% of clusters may be correlated by DYRK1A and SMARCA4. (D) Venn diagram highlighting shared peaks gained by DYRK1A and SMARCA4 complementation. (E) Correlation heatmap comparing changes in RNA abundance in DYRK1A or SMARCA4 complemented cells, identifying a correlation coefficient of 0.08 supporting <10% of the top up-regulated/down-regulated genes are shared between DYRK1A and SMARCA4. (F) Gene set enrichment analysis from RNA-Seq experiments showing shared pathway regulation by DYRK1A and SMARCA4. Data underlying this figure can be found under GEO Accessions GSE213999 and GSE186201. DYRK1A, Dual Specificity Tyrosine Phosphorylation Regulated Kinase 1A; KO, knockout; WT, wild-type. (PDF) [file pbio.3002097.s006.pdf]

Supplemental Figure 5

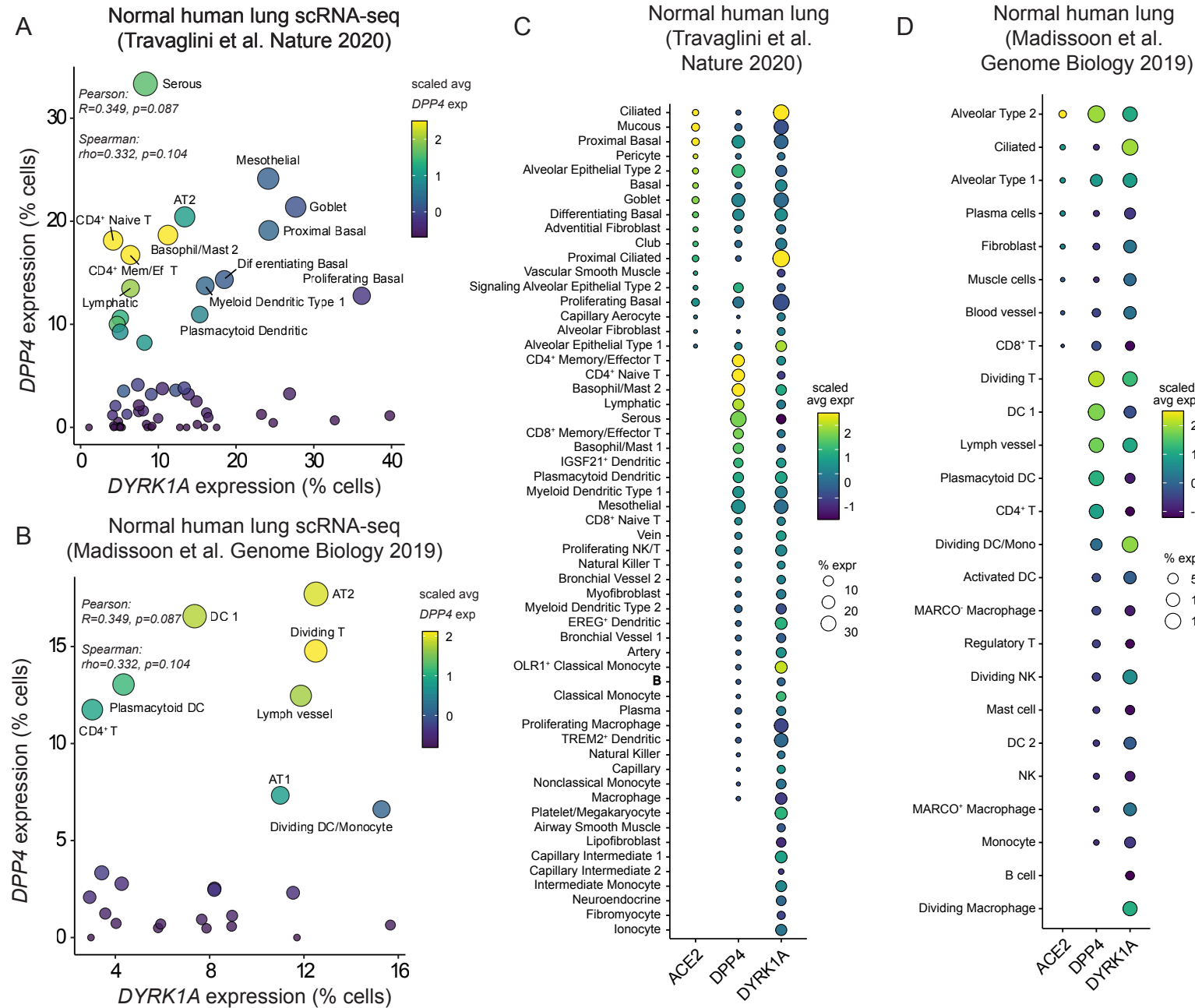

Supplement: S5 Fig — (A, B) Scatter plots and (C, D) dot plots assessing scaled average expression of DYRK1A and (A-E) DPP4 or (C, D) ACE2 from existing scRNA-seq datasets [94,95]. Data in (A. B) were analyzed by Pearson and Spearman correlation statistical tests. ACE2, angiotensin-converting enzyme 2; DPP4, dipeptidyl peptidase-4; DYRK1A, Dual Specificity Tyrosine Phosphorylation Regulated Kinase 1A; scRNA-seq, single-cell RNA sequencing. (PDF) [file pbio.3002097.s007.pdf]

Supplemental Figure 6

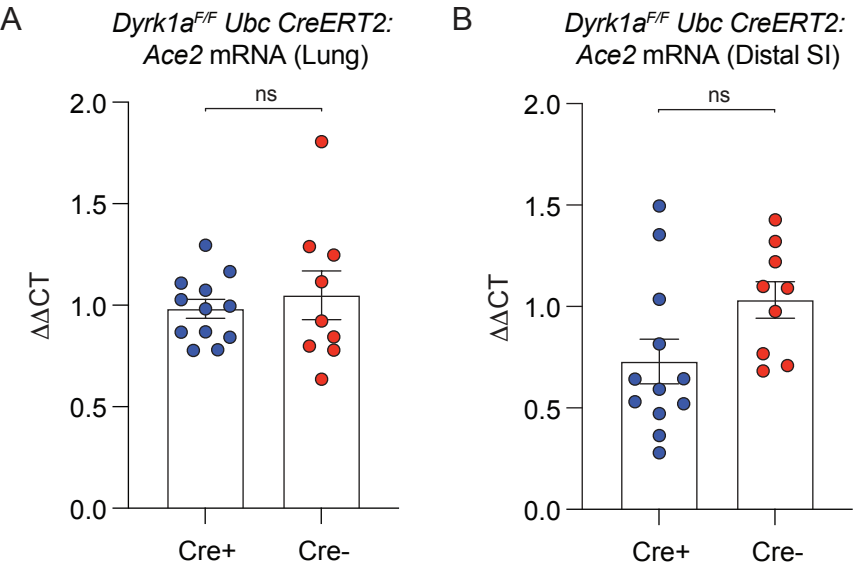

Supplement: S6 Fig — DYRK1A was conditionally deleted by treating Dyrk1aAF/F Ubc CreERT2 mice with tamoxifen for 5 consecutive days. On day 6, (A) lung and (B) distal small intestine homogenates were assessed by RT-qPCR for Ace2 expression. Each data point represents an individual mouse. Error bars represent ± SEM, and statistical comparisons were generated via Student t test; ns p > 0.05. Each experiment was performed 2 independent times with at least 3 mice per group. Shown the ΔΔCT values for each mouse normalized to actin and Cre negative controls. Data underlying this figure can be found in S1 Data. ACE2, angiotensin-converting enzyme 2; DYRK1A, Dual Specificity Tyrosine Phosphorylation Regulated Kinase 1A; RT-qPCR, quantitative reverse transcription PCR. (PDF) [file pbio.3002097.s008.pdf]
